# Transcriptome analysis uncovers the gene expression profile of salt-stressed potato (*Solanum tuberosum* L.)

**DOI:** 10.1038/s41598-020-62057-0

**Published:** 2020-03-25

**Authors:** Qing Li, Yuzhi Qin, Xinxi Hu, Guangcun Li, Hongying Ding, Xingyao Xiong, Wanxing Wang

**Affiliations:** 1 Institute of Vegetables and Flowers, Chinese Academy of Agricultural Sciences/Key Laboratory of Biology and Genetic Improvement of Root and Tuber Crops, Ministry of Agriculture and Rural Affairs, Beijing, 100081 China; 2 College of Horticulture, Hunan Agricultural University/Hunan Provincial Engineering Research Center for Potatoes/Southern Regional Collaborative Innovation Center for Grain and Oil Crops in China, Changsha, 410128 China

**Keywords:** RNA sequencing, Salt

## Abstract

Potato (*Solanum tuberosum* L.) is an important staple food worldwide. However, its growth has been heavily suppressed by salt stress. The molecular mechanisms of salt tolerance in potato remain unclear. It has been shown that the tetraploid potato Longshu No. 5 is a salt-tolerant genotype. Therefore, in this study we conducted research to identify salt stress response genes in Longshu No. 5 using a NaCl treatment and time-course RNA sequencing. The total number of differentially expressed genes (DEGs) in response to salt stress was 5508. Based on Gene Ontology (GO) and Kyoto Encyclopedia of Genes and Genomes (KEGG) analysis, it was found that DEGs were significantly enriched in the categories of nucleic acid binding, transporter activity, ion or molecule transport, ion binding, kinase activity and oxidative phosphorylation. Particularly, the significant differential expression of encoding ion transport signaling genes suggests that this signaling pathway plays a vital role in salt stress response in potato. Finally, the DEGs in the salt response pathway were verified by Quantitative real-time PCR (qRT-PCR). These results provide valuable information on the salt tolerance of molecular mechanisms in potatoes, and establish a basis for breeding salt-tolerant cultivars.

## Introduction

Salt is a major abiotic factor affecting plant growth and secondary metabolism^1^. Soil salinization has become a global problem with about 8 × 10^8^ hectares of soil worldwide threatened by salinization^2^. Salinity interferes with plant growth as it leads to physiological drought and ion toxicity^3^. In addition, other secondary stresses, such as oxidative damage, can occur in plants subjected to high NaCl concentrations^1^. With the increase of salinization, it is a tough challenge to increase grain output and achieve food security.

Potato (*Solanum tuberosum* L.) is an extremely important food staple worldwide due to its versatility and nutritional value. However, potato is quite sensitive to salt stress, which is one of the most important factors limiting its cultivation^4^ and which can lead to serious declines in yield^5,6^. Therefore, there is a great need to improve the salt tolerance of potato and breed salt-tolerant varieties. What’s more, illuminating the molecular mechanisms underlying salt tolerance and identifying the related genes of tolerant plants may contribute to further understanding the functions of these unique genes.

Previous studies have revealed mechanisms underlying salt stress tolerance in plants. Plant membrane receptors sense extracellular salt stress stimuli, and then these stimuli signals are translated into intracellular signals through the generation of second messengers such as calcium, reactive oxygen species (ROS) and inositol phosphates. These second messengers then activate transcription factors (TFs) or protein kinases (PKs), inducing specific genes to be differentially expressed^7^. These signal cascades result in the expression of multiple stress-responsive genes, the products of which can directly or indirectly confer stress tolerance^8^. The complex gene expression cascades activated during the response to salt stress involve signaling pathways related to Na^+^ efflux and Na^+^ localization^9^, which are regulated by calcium-activated 14-3-3 proteins that act as molecular switches^10^. In addition, the mitogen-activated protein kinases (MAPKs) and SOS (salt overly sensitive) can jointly participate in Na^+^ efflux and Na^+^ localization signaling pathways to promote the maintenance of ion equilibrium^9^. Therefore, receptor proteins, Ca signals, TFs and PKs play pivotal roles in salt stress responses.

The SOS signaling pathway is the most widely studied salt tolerance pathway in plants at present^11^. The regulation of salt stress response by the SOS pathway mainly involves five core components, the calcineurin B-like protein 1/10 (SCaBP5/8)/CBL-interacting serine/threonine-protein kinase 24 (SOS2) complex, the CBL-interacting serine/threonine-protein kinase 5 (PKS5)/14-3-3 protein complex, SOS1 (a plasma membrane Na^+^/H^+^ antiporter), AHX (a vacuolar Na^+^/H^+^ exchanger) and ATPase^12^. Under normal conditions, PKS5 or PKS24 phosphorylates SOS2, promotes the binding of SOS2 to 14-3-3 and inhibits the activity of SOS2^13^. At the same time, these kinases inhibit the activity of ATPase on the membrane^12^. Under salt stress, the annexin (ANN) protein mediates the production of an early transient Ca^2+^ signal and leads to an increased Ca^2+^ level in a short period of time^14^. When Ca^2+^ reaches a certain concentration, Ca^2+^ binds to 14-3-3 and ScaBP5/8, which causes 14-3-3 to dissociate from SOS2^10^ and bind to PKS5/24, inhibiting its kinase activity with the help of J3 (DnaJ homolog3) and eliminating the inhibition of SOS2 and ATPase^15^. In addition, SCaBP5/8 binds the activated SOS2, and the SCaBP5/8-SOS2 complex inhibits ANN activity, maintaining the intracellular Ca^2+^ concentration at a certain level^14^. Plasma membrane type H^+^-ATPase and vacuolar H^+^-ATPase are activated by SOS2, which establishes a proton gradient across the plasma or vacuolar membrane that drives SOS1 or NHX activity to promote cellular ion equilibrium^9^. In addition to the well characterized ANN–14-3-3–PKS5/24/J3–SCaBP5/8–SOS2–SOS1 signaling pathway and ANN–14-3-3–PKS5/24/J3–SCaBP5/8–SOS2–NHXs signaling pathway, there are still networks that are as yet uncharacterized.

So far, a few salt-responsive genes have been identified in potato, such as zinc finger protein gene (*StZFP1)*^16^; ethylene-responsive transcription factors (*StERF1, StERF3* and *StERF6)*^17^*;* dehydration-responsive element-binding protein genes (*StDREB1, StDREB2, StDREB3* and *StDREB4)*^17,18^. With the development of high throughput sequencing technology, RNA-seq has been more and more frequently used in plant stress resistance research. Meanwhile, the release of the potato reference genome sequence has provided the opportunity to identify salt-tolerance genes genome-wide in potato^19^. For example, expression profiling of the NAC transcription factor family in potato using RNA-seq disclosed that *StNAC024, StNAC067* and *StNAC108* are induced specifically under salt stress^20^. However, there are still insufficient expression data for revealing the molecular mechanisms of potato salt tolerance^21^. Isolating salt-tolerance genes and understanding the molecular mechanisms of salt tolerance are important for effectively improving salt tolerance in the existing cultivated potato species. Although sensitivity to salt is a characteristic of potato^5^, salt-tolerant tetraploid potato germplasm resources do exist^22^. For example, in a resource identification and evaluation study, we identified a tetraploid potato genotype, Longshu No. 5, with strong salt tolerance^23^. NaCl is most widely used to induce salt stress because of its higher solubility^24,25^. Hence, we subjected Longshu No. 5 to NaCl-induced salt stress and used RNA-seq to research the changes in the gene expression profiles to explore the salt-tolerance molecular mechanisms in potato and provide a basis for breeding salt-tolerant varieties.

## Results

### Transcriptome sequencing, assembly, and mapping

**Figure 1:**
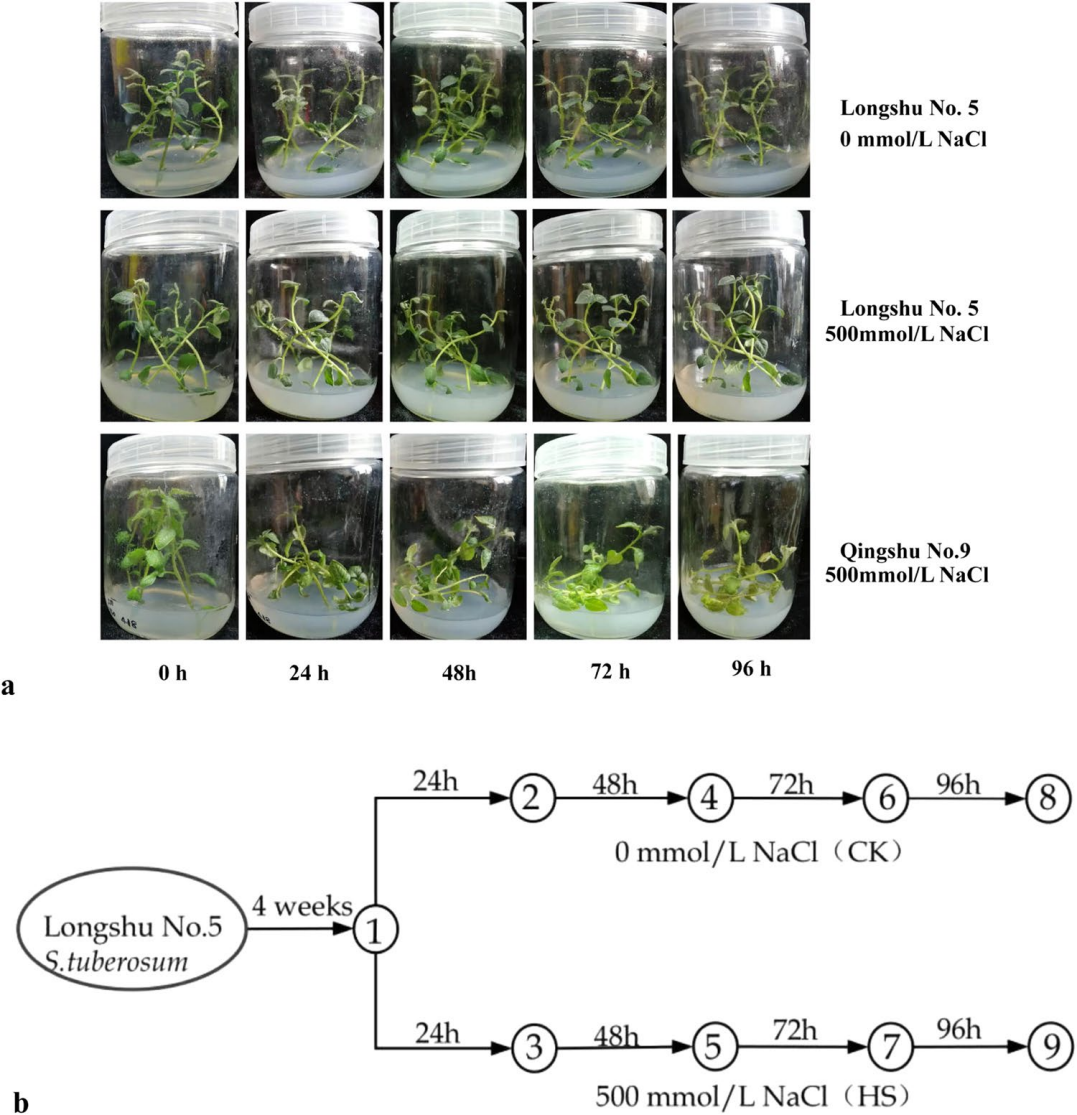
Material and schematic of the experimental design. (**a**) Phenotypes of Longshu No. 5 treated with 500 mmol/L NaCl (HS) or 0 mmol/L NaCl (CK) for 0, 24, 48, 72 and 96 h. Qingshu No. 9 was salt-sensitive control. (**b**) Schematic showing the experimental design and nine sampling points for Longshu No. 5. Plants were collected from three replicates at each time point, and point ① is Longshu No. 5 at the 0 h time point.

The tetraploid potato genotype Longshu No. 5 seedlings were treated with 500 mmol/L NaCl in the salt-stress experiment. Except for slight leaf rolling, which was observed after 48 h of NaCl stress, Longshu No. 5 looked similar to control seedlings grown without NaCl, while severe lodging and wilting were observed in the salt-sensitive genotype Qingshu No. 9 after 24 h (Fig. 1a). To obtain comprehensive gene expression information for the salt-stress response in *S. tuberosum*, we analysed the transcriptome profile of Longshu No. 5 (4 weeks seedling age) grown in tissue medium containing 500 mmol/L NaCl (High salt group, HS) or 0 mmol/L NaCl (Control group, CK) for 24, 48, 72, and 96 h (Fig. 1b).

RNA was extracted from the salt-treated seedling samples (HS groups; 24, 48, 72 and 96 h) and the untreated samples (CK groups; 0, 24, 48, 72 and 96 h) with three replicates per time point (total of 27 samples). The extracted RNA was verified to be of good quality (Supplementary Fig. S1**;** Supplementary Table S1). Subsequently, 27 libraries were constructed and sequenced using the Illumina HiSeqX10 platform with the 150-cycle paired-end sequencing protocol^26^. Through filtering raw data and assessing quality, an average of 11.74 G clean bases (77.89 million paired-end reads) per sample were obtained. The Q30 percentage (the proportion of bases with a quality value greater than 30) was 95%–97%, and more than 84.4% of the RNA-seq reads could be mapped to the DM reference genome (Supplementary Table S2).

### Differential gene expression in response to NaCl treatment

**Figure 2:**
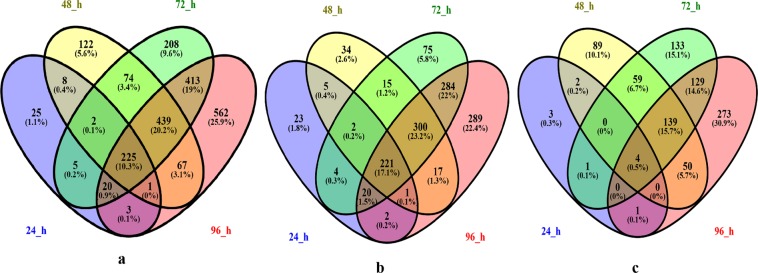
A venn diagram showing the overlap in differentially expressed genes (DEGs) under 24, 48, 72 and 96 h of NaCl stress in Longshu No. 5. (**a**), (**b**), and (**c**) are the Venn diagrams for all, up-regulated, and down-regulated genes, respectively. Due to PGSC0003DMG400004090 down-regulated at 24 h and up-regulated at 48 h, the numbers of up- and down-regulated genes don’t match the total number of genes at 24 h and 48 h.

Fragments per kilobase of transcript per million mapped reads (FPKM) generated using DESeq. 2 was used as the measure of gene expression. An absolute log2 (fold change) value ≥1 and a false discovery rate (FDR) < 0.01 were set as the threshold for significant differential expression. Pairwise comparisons were performed between each HS group (24 h, 48 h, 72 h and 96 h) and the respective CK group to generate an HS DEG library, and the CK groups (0 h, 24 h, 48 h, 72 h and 96 h) were compared with each other to generate a CK DEG library. In total, 4297 and 5558 DEGs were detected for the CK groups and HS groups, respectively. A total of 5508 DEGs remained after removing genes in the CK-DEG library from the HS-DEGs library. 2810 of which were up-regulated and 2700 of which were down-regulated (the two DEGs of 5508 were up-regulated at some stress time points and down-regulated at others) (Table 1). A total of 289 (278 up-regulated and 11 down-regulated), 938 (595 up-regulated and 343 down-regulated), 1386 (921 up-regulated and 465 down-regulated), and 1730 DEGs (1134 up-regulated and 596 down-regulated) were detected after 24, 48, 72 and 96 h of NaCl stress, respectively (Table 1, Fig. 2). Venn diagram analysis showed that 25 (23 up-regulated and 3 down-regulated), 122 (34 up-regulated and 89 down-regulated), 208 (75 up-regulated and 133 down-regulated) and 562 (289 up-regulated and 273 down-regulated) DEGs were specifically regulated in Longshu No. 5 after 24, 48, 72 and 96 h of NaCl stress, respectively (Fig. 2). A total of 225 DEGs were commonly regulated at 24, 48, 72 and 96 h, including 221 up-regulated and 4 down-regulated genes. A sharp increase in the number of DEGs after 24 h of stress indicated that an adaptive response to NaCl stress was initiated in Longshu No. 5.

**Table 1:** Differentially expressed genes that were specifically up-regulated or down-regulated by NaCl stress in potato genotype Longshu No. 5.

DEG library	Comparison	Number of DEGs	Up-Regulated DEGs	Down-Regulated DEGs
HS DEG library	HS24 vs CK24	461	404	57
	HS48 vs CK48	1701	902	799
	HS72 vs CK72	2189	1394	795
	HS96 vs CK96	2657	1665	992
CK DEG library	CK24 vs CK0	1331	965	366
	CK48 vs CK0	933	654	279
	CK72 vs CK0	1566	840	726
	CK96 vs CK0	2134	1073	1061
	CK24 vs CK48	2112	847	1265
	CK24 vs CK72	4280	1681	2599
	CK24 vs CK96	3399	1905	1494
	CK48 vs CK72	172	113	59
	CK48 vs CK96	609	464	145
	CK72 vs CK96	1097	534	563
Total*		5508	2810	2700

*: the total number of genes specifically differentially expressed in response to NaCl stress after removing duplicate genes.

### GO and KEGG enrichment analysis of DEGs

**Figure 3:**
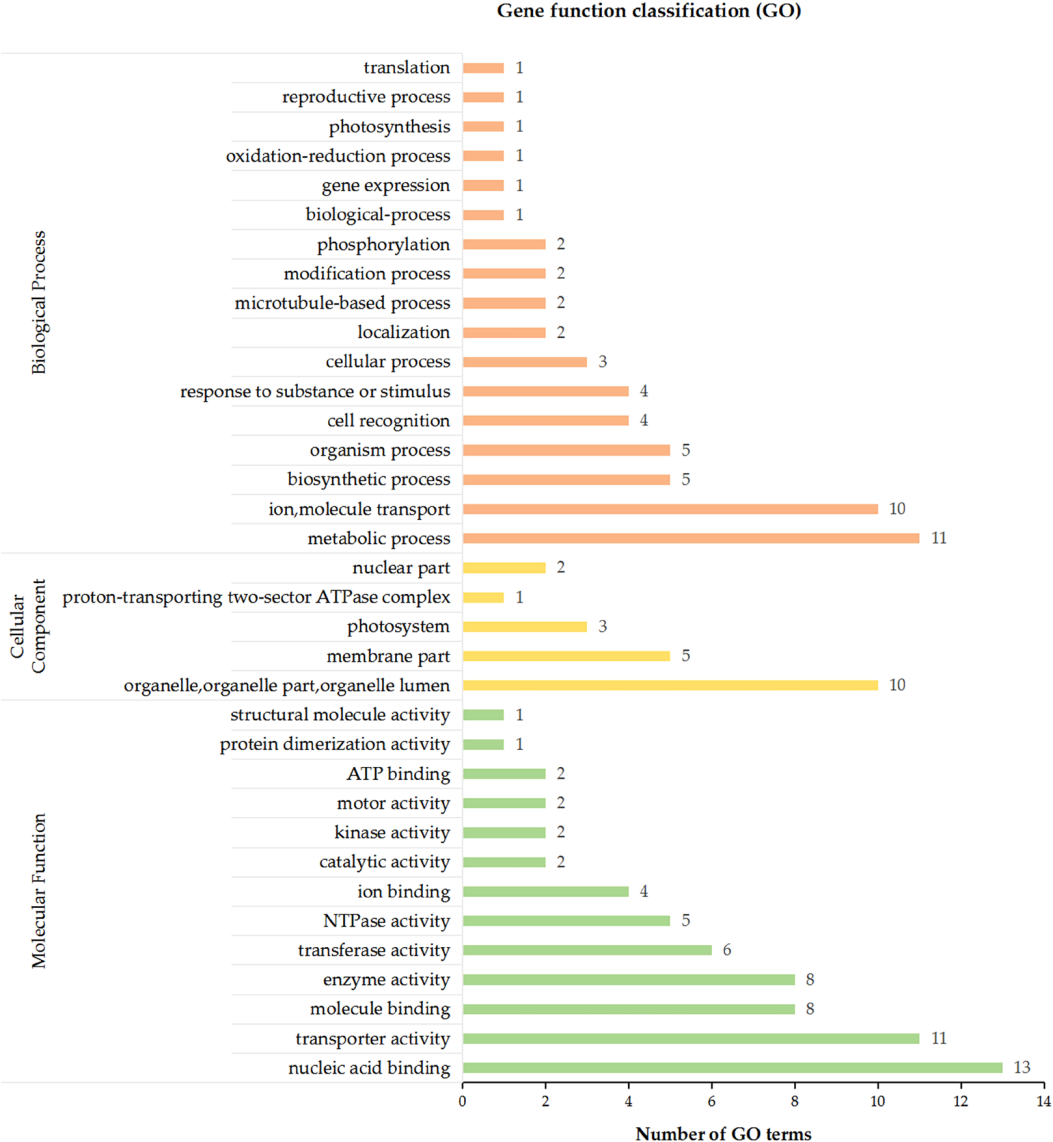
Gene ontology (GO) terms enriched in differentially expressed genes.

Gene ontology (GO) analysis was performed, and 2879 of 5508 DEGs was annotated to one or more GO terms. 142 GO terms that were significantly enriched in DEGs were categorized into 35 groups. Most of the enriched biological process GO terms were linked to metabolic process (11 terms) and ion or molecular transport (10 terms). Most of the enriched molecular function GO terms were related to organelle, organelle part, organelle lumen (10 terms), and the highest number of the DEGs were annotated to membrane (GO: 0016020; 401 DEGs). In the molecular function class, most DEGs including many TFs were annotated to nucleic acid binding (13 terms), followed by transporter activity (11 terms), ion binding (4 terms) and kinase activity (2 terms), which play a crucial role in salt stress response^9^, and a large number of the DEGs were annotated to catalytic activity (GO: 0003824; 1452 DEGs), ion binding (GO: 0043167; 856 DEGs) and transferase activity (GO: 0016740; 613 DEGs) (Fig. 3).

**Figure 4:**
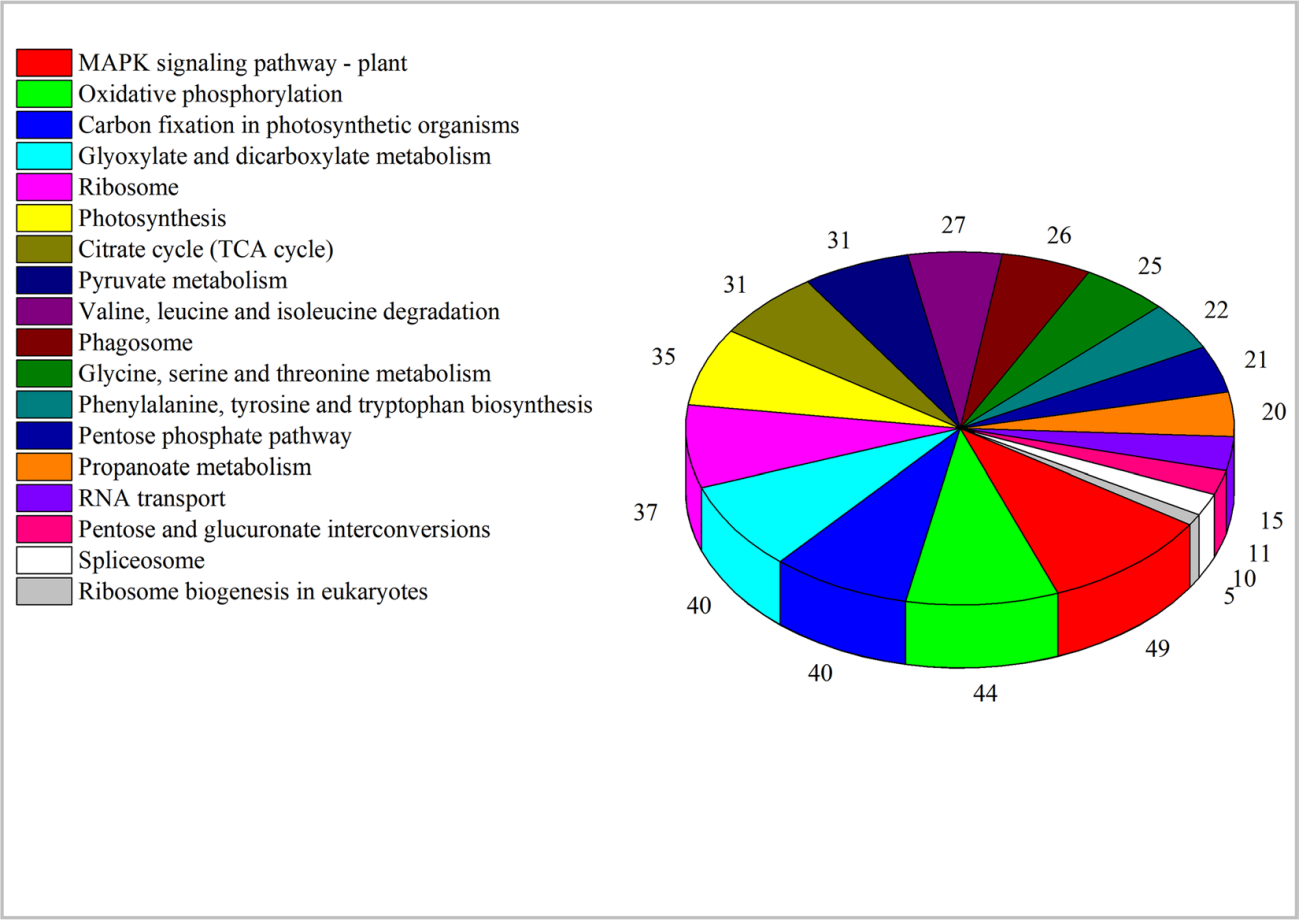
The number of DEGs annotated to the main enriched Kyoto Encyclopedia of Genes and Genomes (KEGG) pathways in Longshu No. 5.

We next performed Kyoto Encyclopedia of Genes and Genomes orthologs (KEGG) analysis; 1760 DEGs had KEGG annotation and were assigned to 137 KEGG pathways. The photosynthesis, carbon fixation in photosynthetic organisms, citrate cycle (TCA cycle), spliceosome, ribosome, glyoxylate and dicarboxylate metabolism categories were the most significantly enriched in DEGs (Supplementary Table S3). In addition, a large number of DEGs were annotated to MAPK signaling pathway-plant, oxidative phosphorylation, carbon fixation in photosynthetic organisms and glyoxylate, and dicarboxylate metabolism (Fig. 4).

### Differential expression of transcription factors in response to NaCl treatment

**Figure 5:**
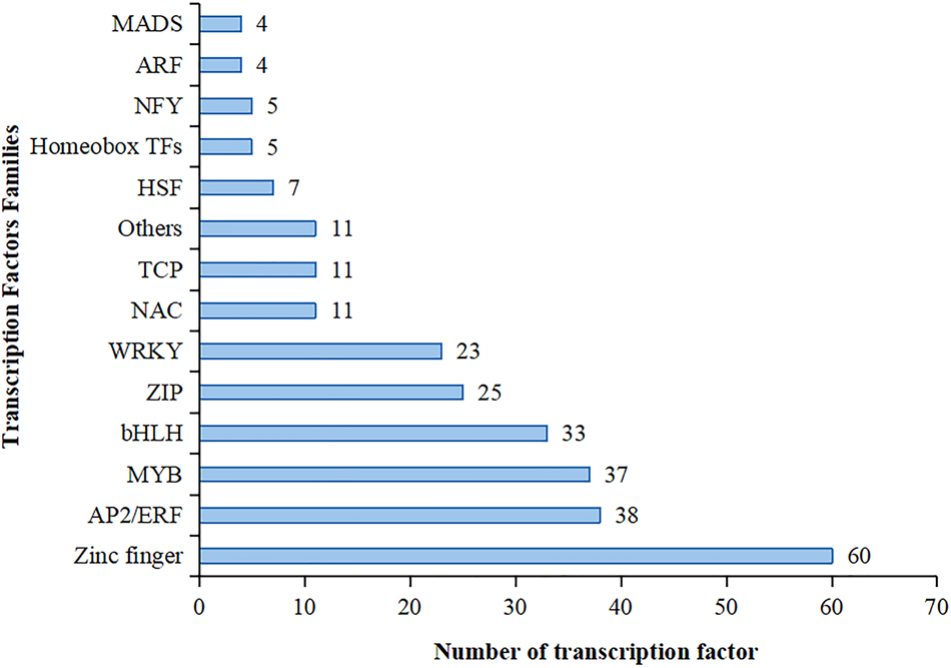
Number of transcription factors in different families differentially expressed under salt stress in potato. Others indicate uncharacteristic proteins with transcription factor activity.

Among the 5508 genes differentially regulated by NaCl stress, 274 encoded TFs belonging to 13 families. Most TFs were zinc finger proteins, followed by AP2-like ethylene-responsive transcription factor (AP2/ERF), MYB, bHLH, ZIP, WRKY, NAC, transcription factor TCP (TCP), heat stress transcription factor (HSF), Homeobox TFs, nuclear transcription factor Y (NFY), auxin response factor (ARF), MADS-box transcription factor (MADS) and others (Fig. 5).

About half of the TFs (53.3%) were up-regulated under salt treatment. Among the 60 zinc fingers proteins, there was an equal number of up-and down-regulated members, of which 10 genes were continuously up-regulated during salt stress. The gene PGSC0003DMG400023518, encoding ZTP2-14, was significantly up-regulated with a fold change of 4.5, 6.1, 8.2, and 8.4 at 24, 48, 72 and 96 h, respectively. Twenty-three of the 38 AP2/ERFs were up-regulated under salt treatment. Among the MYBs family, 19 were up-regulated; the expression of *MYB36* (PGSC0003DMG400018331) and *MYB108* (PGSC0003DMG400004612) continued to increase with prolonged salt stress, with a fold change in expression of 10.0 and 9.87 at 96 hours, respectively. Seventeen members of the bHLH family were up-regulated, especially transcription elongation factor SPT (PGSC0003DMG400004011), whose expression increased more than 4-fold after 72 h of stress. Among the ZIP, WRKY, NAC, TCP, HSF, Homeobox TFs, NFY, ARF, and MADS TF family members, 54 genes were up-regulated under salt stress in potato. Of these, *WRKY45* (PGSC0003DMG400020206), *WRKY61* (PGSC0003DMG400018081), *TGA-2.1* (PGSC0003DMG400023678) and *AGL15* (PGSC0003DMG401006771) were very lowly expressed (FPKM < 0.1) in the untreated group and moderately or highly expressed (FPKM ≥ 3.75) in the salt-treated group. *WRKY7* (PGSC0003DMG400024961) and *WRKY14* (PGSC0003DMG400015104) were continually up-regulated. In addition, *NAC083* (PGSC0003DMG400011891) was highly expressed at 96 hours under NaCl stress with a FPKM value of 754.4. PGSC0003DMG400002484, encoding NFY, was highly expressed after 48 hours. In addition, four ARFs (PGSC0003DMG400012261, PGSC0003DMG400008065, PGSC0003DMG400003771, PGSC0003DMG400008081) down-regulated under NaCl stress (Supplementary Table S4).

### Signal transduction-related proteins differentially expressed in response to NaCl stress

#### Protein kinases differentially expressed under salt stress

**Figure 6:**
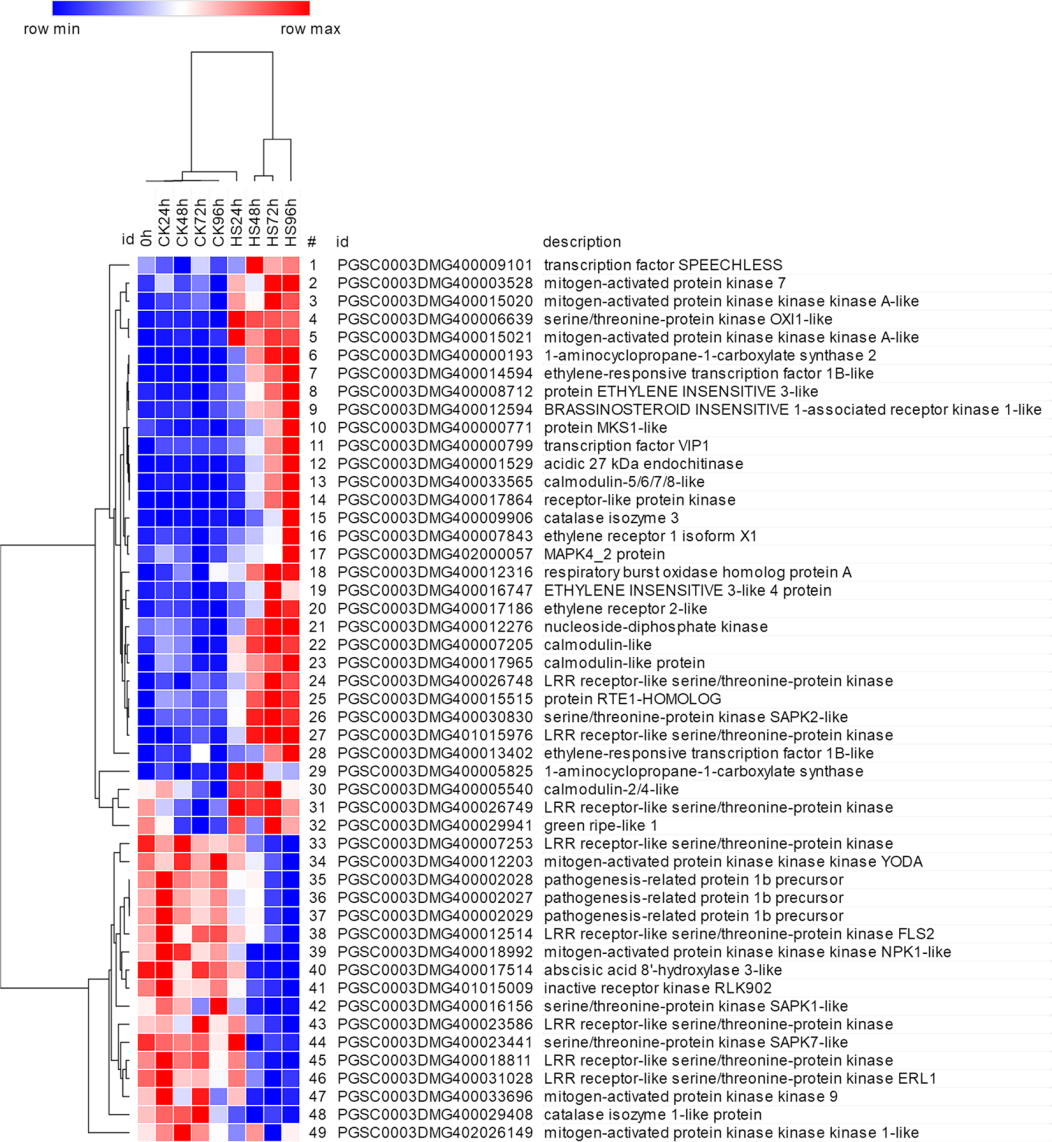
MAPK signal pathway genes were differentially expressed in response to salt stress in potato. DEGs 1–32 were up-regulated, and DEGs 33–49 were down-regulated.

PKs act as a signal transducer or receptor protein and play a crucial role in phosphorylation events. We identified 259 PK genes with a moderate or high level of expression (FPKM ≥ 3.75), and many of them (124) encoded receptor-like kinases (RLKs), including LRR receptor-like kinases (LRR-RLKs, 46 members) and receptor-like protein kinases (RPKs, 26 members). LRR-RLKs and RPKs occupied 58.1% of RLKs. Besides, lots of serine/threonine protein kinases (STPKs) family were induced by salt stress, and 77.8% of CBL-interacting protein kinases (CIPKs) were all up-regulated (Supplementary Table S5). Mitogen-activated protein kinase families (MAPKs) play a crucial role in MAPK signaling pathways. There were 49 DEGs involved in the MAPK signaling pathway, of which 32 genes were up-regulated and 17 genes were down-regulated. For example, DEGs encoding an RPK (PGSC0003DMG400017864), a calmodulin-like protein (PGSC0003DMG400033565), an ethylene response transcription factor (PGSC0003DMG400014594), an ethylene receptor (PGSC0003DMG400017186), a catalase isoenzyme (PGSC0003DMG400009906), and a mitogen-activated protein kinase kinase kinase (MAPKKK) gene (PGSC0003DMG400015021), were up-regulated by 8.51-, 7.63-, 5.51-, 4.08-, 3.37- and 3.09-fold, respectively, after 96 hours of salt stress. Transcription factor VIP1 (PGSC0003DMG400000799), which is involved in the MAPK signaling pathway, was previously shown to be induced by osmotic stress^27^ and it was also up-regulated in potato under salt stress. All three genes (PGSC0003DMG400002028, PGSC0003DMG400002029 and PGSC0003DMG400002027) encoding the pathogenesis-related protein 1b precursor, which is involved in the MAPK pathway, were down-regulated (Fig. 6**;** Supplementary Table S6).

#### Ca signaling pathway-related genes differentially expressed under salt stress

A total 53 DEGs belonging to 14 families related to Ca signal were induced by salt stress. About half of the DEGs (26 genes) encoded calcium-transporting ATPases (ACAs), CIPKs or calcium-binding proteins (CMLs). Of these, *CIPK11* (PGSC0003DMG400020564), *CIPK14* (PGSC0003DMG400011106, PGSC0003DMG400022019) and *CIPK24* (PGSC0003DMG400006384) were continually up-regulated with increasing duration of salt stress, and there was a large change in the expression level of *CML19*, with fold-change values of 3.93-, 4.33-, 5.92- and 9.64-fold at 24, 48, 72 and 96 hours under salt stress, respectively. In addition, 19 DEGs encoding calmodulin-like proteins (CALMs), calmodulin-binding receptor-like cytoplasmic kinases (CRCKs), calcineurin B-like proteins (CBLs), calmodulin-binding proteins (CBPs), calcium-dependent protein kinases (CDPKs), calcium permeable stress-gated cation channels (CSCs), calmodulin-binding transcription activators (CAMTAs), calcium homeostasis regulators (CHoRs), calcium sensing receptors (CASs), calcium-binding mitochondrial carrier proteins (SCaMCs) and two pore calcium channel proteins (TPCNs) were up-regulated in response to salt stress. *CRCK2* (PGSC0003DMG402006188), *CBP60C* (PGSC0003DMG400024785) and *CBL10* (PGSC0003DMG400029942) were expressed at particularly high levels with FPKM values of 11042.33, 9438.67 and 7547.67, respectively, at 96 hours under salt stress (Table 2).

**Table 2:** Genes encoding Ca signal pathway-related proteins differentially expressed in response to salt stress in potato.

Ca signal pathway-related proteins	Number	Known genes*
Calcium-transporting ATPases, ACAs	9	*ACA2, ACA10, ACA12*
CBL-interacting protein kinases, CIPKs	9	*CIPK3, CIPK11, CIPK14, CIPK18, CIPK24, CIPK2, CIPK5*
Calcium-binding proteins, CMLs	8	*CML19, CML44, CML25, KIC*
Calmodulin-likes, CALMLs	5	*CALML5*
Calmodulin-binding receptor-like cytoplasmic kinases, CRCKs	4	*CRCK2*
Calcineurin B-like proteins, CBLs	3	*CBL1, CBL10, CBL7*
Calmodulin-binding proteins, CBPs	3	*CBP60C*
Calcium-dependent protein kinases, CDPKs	3	*CPK11*
Calcium permeable stress-gated cation channels, CSCs	3	*—*
Calmodulin-binding transcription activators, CAMTAs	2	*—*
Calcium homeostasis regulators, CHoRs	2	*CHoR1*
Calcium sensing receptors, CASs	1	*CAS*
Calcium-binding mitochondrial carrier proteins, SCaMCs	1	*—*
Two pore calcium channel proteins, TPCNs	1	*—*

—: no known function in other plants. *: only known function genes in other plants are listed here.

### Stress-related protein genes differentially expressed in response to salt stress

#### Osmoregulation and carbohydrate metabolism-related genes under salt stress

**Figure 7:**
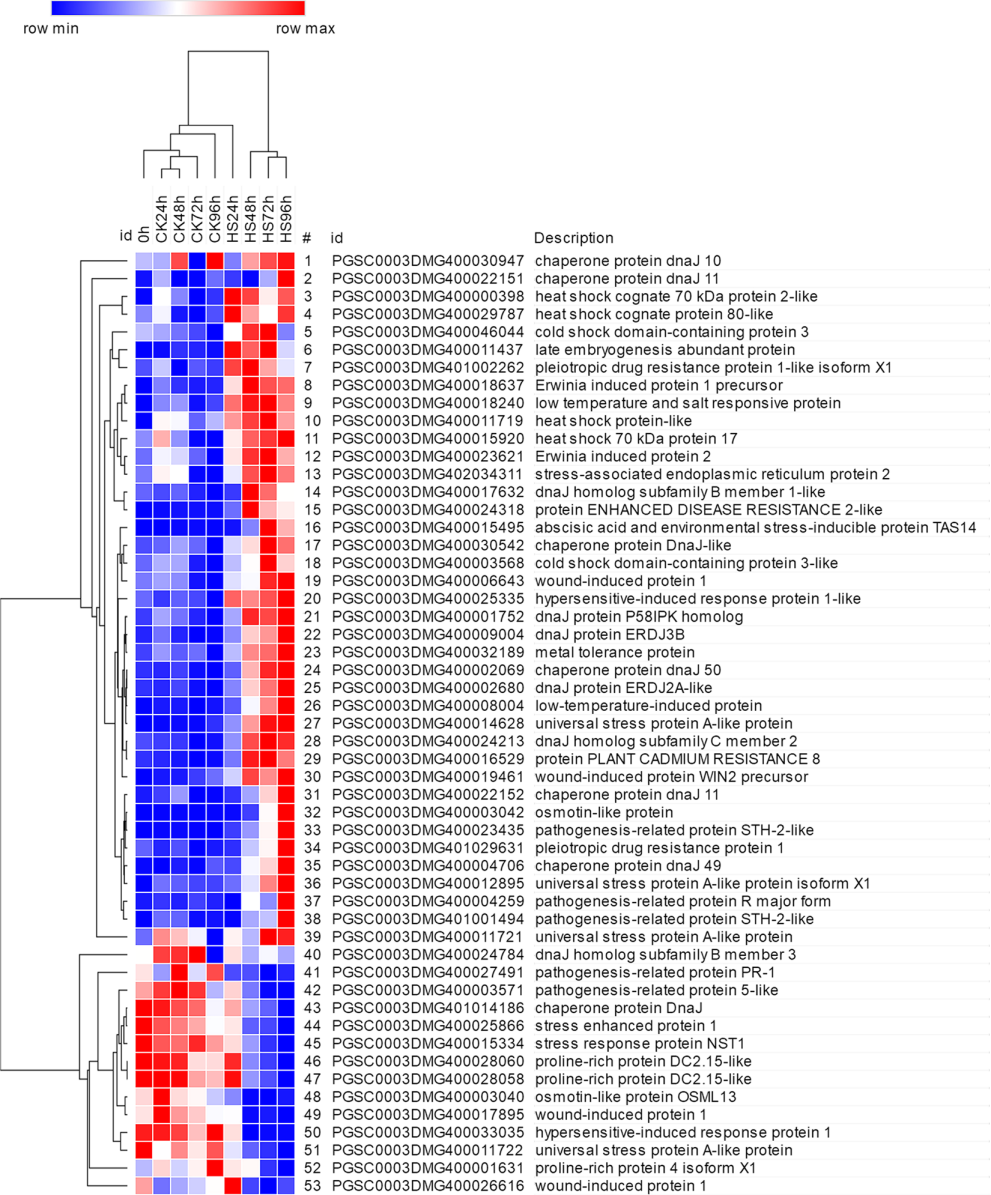
Stress-induced protein genes differentially expressed in response to salt stress. DEGs 1–40 were up-regulated, and DEGs 41–53 were down-regulated.

Stress-induced proteins and carbohydrate metabolism-related proteins can participate in osmotic adjustment. Forty of the 53 DEGs encoding stress-induced proteins were up-regulated under salt stress with relatively high expression observed under stress (FPKM ≥ 3.75), such as chaperone proteins DnaJ, osmotin-like protein, proline-rich proteins (PRPs) and low-temperature and salt-responsive proteins (LTSRs) (Fig. 7). There were 13 DEGs encoding the chaperone protein DnaJ family and 92.3% of them were up-regulated. Pathogenesis-related protein STH-2-like (PGSC0003DMG400023435), abscisic acid and environmental stress-inducible protein TAS14 (PGSC0003DMG400015495), and osmotin-like protein (PGSC0003DMG400003042) were a large change in the expression level, with fold-change values of 8.06-, 7.79- and 7.04-fold at 96 hours under salt stress, respectively. In addition, LTSR (PGSC0003DMG400018240), encoded low temperature and salt responsive protein, was continually up-regulated with prolonged salt stress (Fig. 7). Eighteen DEGs encoded carbohydrate metabolism-related proteins and 69.6% of them were up-regulated. Twelve DEGs encoding UDP-glycosyltransferases (UGTs) were all up-regulated under salt stress, such as *UGT73D1* and *UGT91C1* were up-reguated by 6.38- and 3.13-fold at 96 hours, respectively; PGSC0003DMG400015579, encoding UDP-glycosyltransferase 74B1-like, was positively regulated by salt stress with fold-change values of 3.69, 3.42, 4.27 and 5.20 at 24 h, 48 h, 72 h and 96 h, respectively (Supplementary Table S7).

#### Antioxidant-related genes differentially expressed in response to salt stress

**Figure 8:**
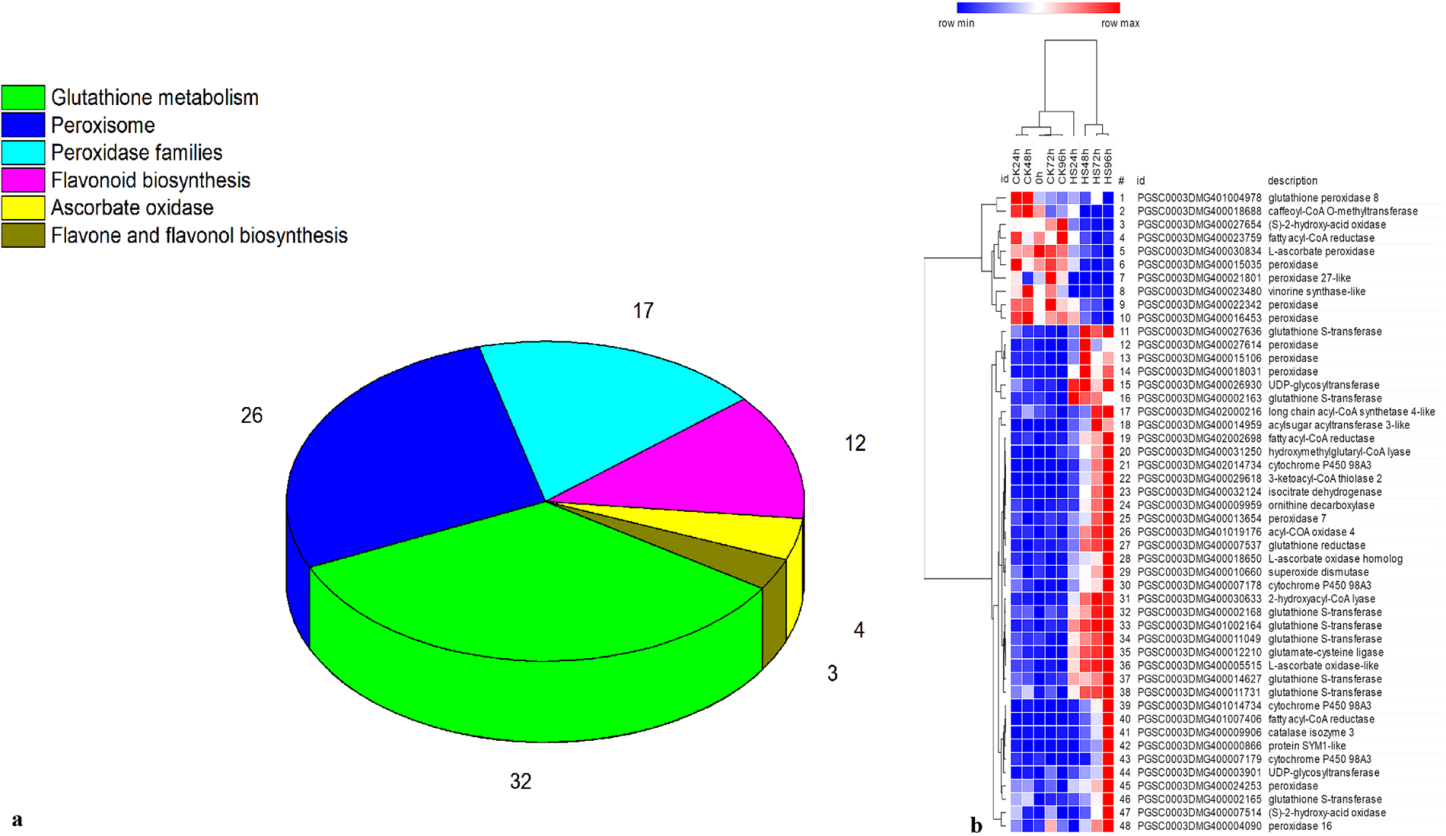
Antioxidant pathway-related genes were differentially expressed in response to salt stress. (**a**) the number of DEGs was related to antioxidant pathways. (**b**) DEGs 1–10 were down-regulated, and DEGs 11–48 were up-regulated.

Sixty-one of the 87 DEGs related to antioxidant pathways were up-regulated and 26 were down-regulated under salt stress. A single DEG could be involved in one or more antioxidant pathways. Most of the DEGs encoded proteins related to glutathione metabolism (32 members), followed by peroxisome (26 members), peroxidase families (17 members), flavonoid biosynthesis (12 members), ascorbate oxidase (4 members) and flavone and flavonol biosynthesis (3 members) (Fig. 8a). Forty-eight out of the 87 DEGs had a large fold change in expression (|Log2 fold change | ≥2). These genes encoded peroxidase (POD), superoxide dismutase (SOD), catalase isozyme 3 (CAT3), glutathione peroxidase 8 (GPX8), cytochrome P450 98A3 (CYP98A3), fatty acyl-CoA reductase 1-like (FARL1), glutathione S-transferase (GST), (S)−2-hydroxy-acid oxidase (HAO), UDP-glycosyltransferase (UGT), vinorine synthase-like (VSL), 2-hydroxyacyl-CoA lyase (HACL1), glutamate-cysteine ligase (GCSA), glutathione reductase (GSR), 3-ketoacyl-CoA thiolase 2 (KAT2), 2-hydroxyacyl-CoA lyase (HCAL), acyl-COA oxidase 4 (ACOX4), caffeoyl-CoA O-methyltransferase (CAMT), hydroxymethylglutaryl-CoA lyase (HMGCL), acylsugar acyltransferase 3-like (AATL3), isocitrate dehydrogenase (IDH), L-ascorbate oxidase-like (AOXL), L-ascorbate oxidase homolog (AOXH), long chain acyl-CoA synthetase 4-like (ACSL4), ornithine decarboxylase (ODC), and protein SYM1-like (PSL) (Fig. 8b). PGSC0003DMG400018031, encoding a lignin-forming anionic peroxidase-like protein, and PGSC0003DMG401007406, encoding a fatty acyl-CoA reductase 1-like protein, were up-regulated and with high fold-change values of 9.87 and 9.07, respectively. *CAT3* (PGSC0003DMG400009906), *SOD1* (PGSC0003DMG400010660), period circadian protein homolog genes *PER7* (PGSC0003DMG400013654) and *PER66* (PGSC0003DMG400024253) were up-regulated at all time points under salt stress.

#### Oxidative phosphorylation-related genes differentially expressed in response to salt stress

**Figure 9:**
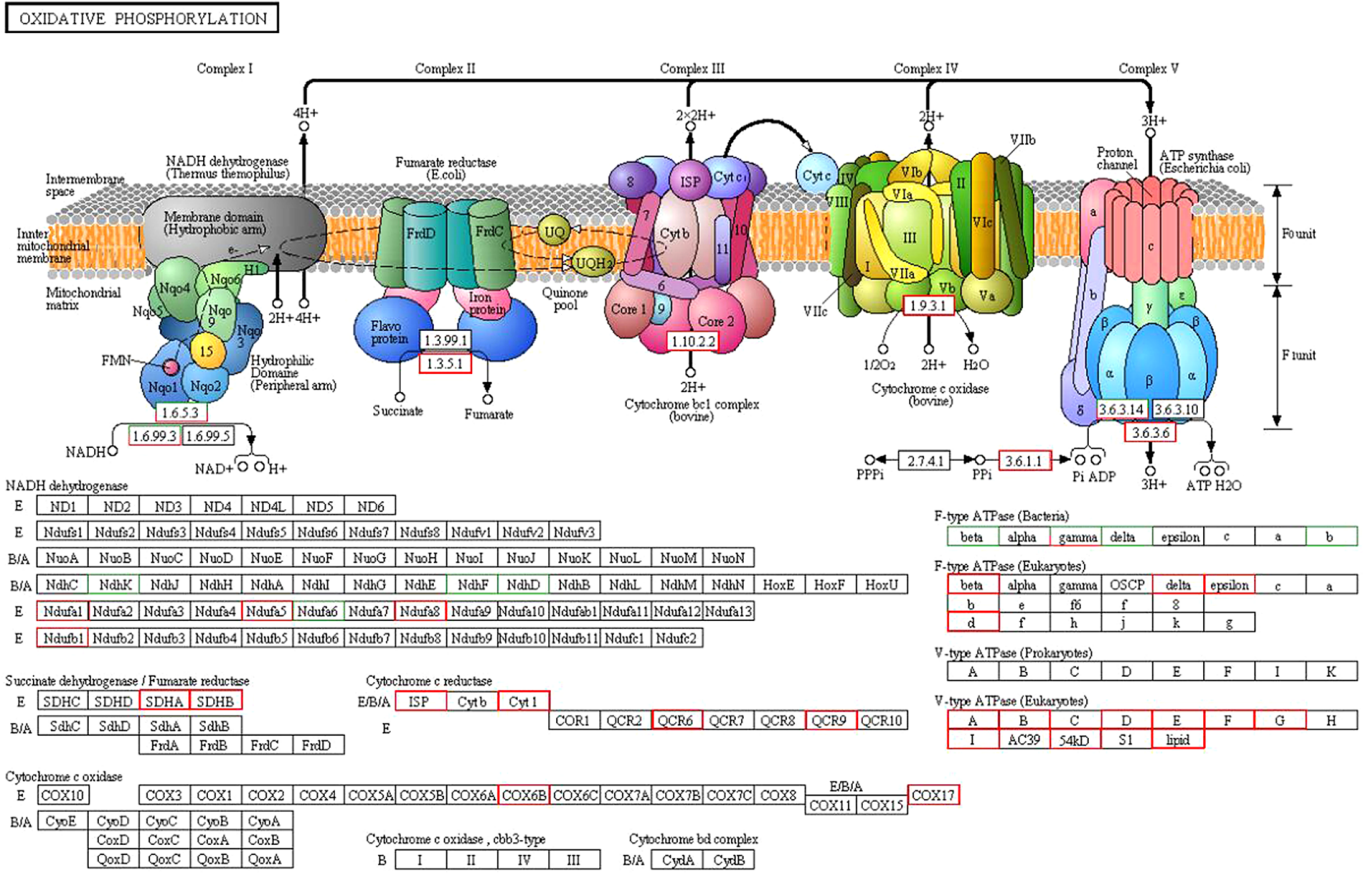
Changes in the expression levels of genes related to the oxidative phosphorylation pathway. Green rectangle represents down-regulation; red rectangle represents up-regulation.

Thirty-four of the 44 DEGs involved in oxidative phosphorylation under salt stress were up-regulated and 10 were down-regulated. These DEGs encoded members of five oxidative phosphorylation complexes; 9 V-type and 4 F-type H^+^ transport ATPases, 2 cytochrome C oxidases, (COX17 and COX6B), the cytochrome reductases (ISP, Cyt1, QOR6 and QCR9), the succinate dehydrogenases (SDHA and SDHB) were up-regulated under salt stress in potato. The inorganic pyrophosphatase gene PGSC0003DMG400002775 was highly differentially expressed at each stress time point, with more than a 5-fold change in expression at 24 h. In addition, The NADH dehydrogenases Ndufa5 and Ndufa8 were also up-regulated, but cytochrome c oxidase subunit (Ndufa6), NAD(P)H-quinone oxidoreductase subunit (NdhK, NdhF and NdhD) were down-regulated with salt stress (Fig. 9). These DEGs indicated that oxidative phosphorylation was response to salt stress in potato.

### Differential expression of ion transport-related genes under salt stress

**Figure 10:**
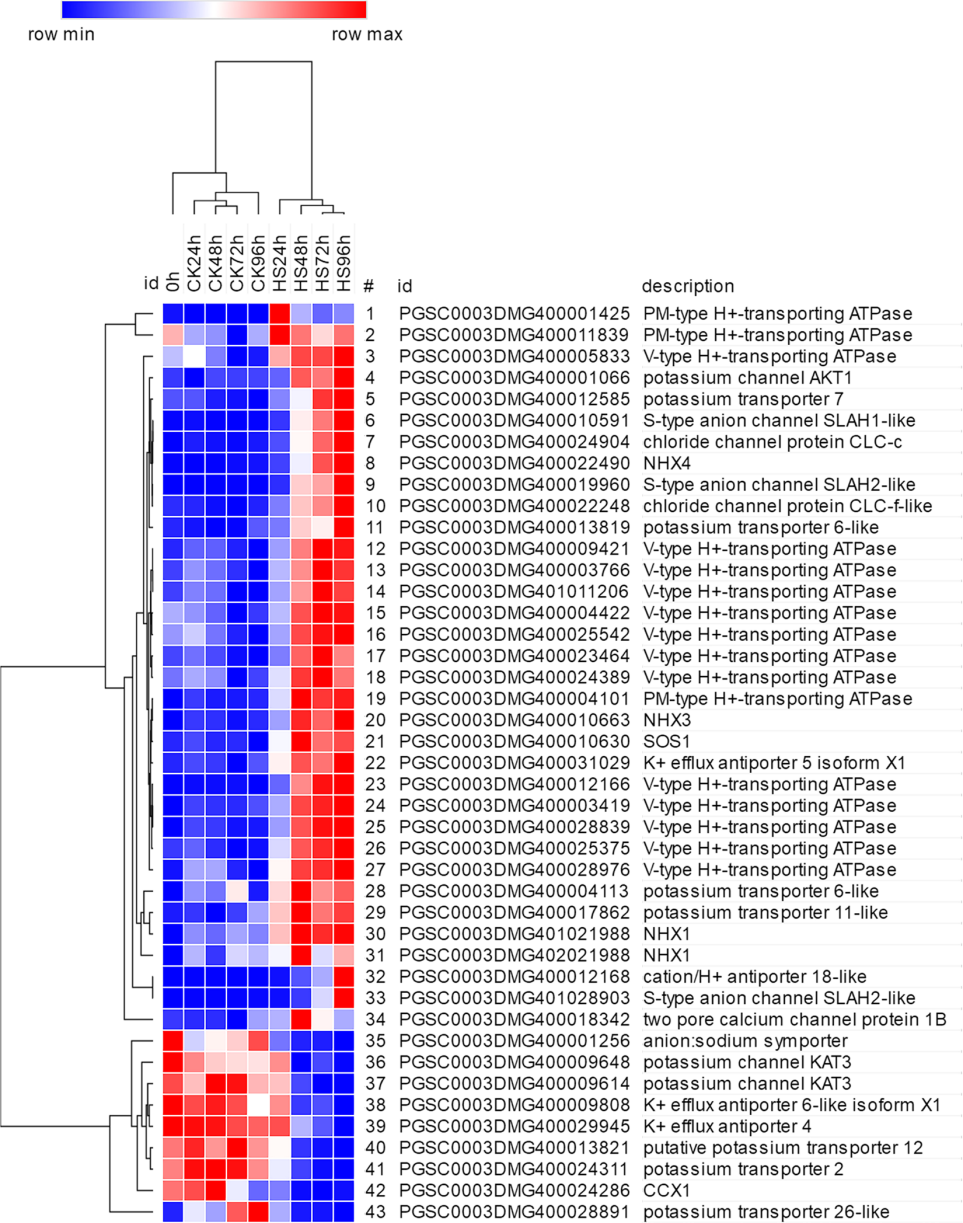
Differential expression of ion transport-related genes under salt stress. DEGs 1–34 were up-regulated, and DEGs 35–43 DEGs were down-regulated.

Forty-three DEGs encoded ion adjustment-related proteins, including potassium transporters (KTs), sodium/hydrogen exchangers (NHEs), K^+^ efflux antiporters (KEAs), SLAH S-type anion channels, chloride channel proteins, potassium channel proteins, anion: sodium symporters, cation/H^+^ antiporters (CHXs), two pore calcium channel proteins (TPCNs), PM-type H^+^ -transporting ATPases (plasma membrane type, PMAs), V-type H^+^-transporting ATPases (vacuole type, ATPeVs) and V-type proton ATPase catalytic subunits. Among them, 34 were up-regulated under salt stress. PGSC0003DMG400004101, which encodes PMA, was the most highly expressed with FPKM values of more than 1000. PGSC0003DMG400012168, which encodes a cation/H^+^ antiporter, was highly differentially expressed with an 8.95-fold change in expression at 96 h. We also found four DEGs (PGSC0003DMG400022490, PGSC0003DMG400010663, PGSC0003DMG401021988, PGSC0003DMG402021988) encoding sodium/hydrogen antiporters (NHX4, NHX3 and NHX1), the key proteins exporting Na^+^ to vacuoles. The expression of these NHX genes increased gradually with extended duration of NaCl stress. In addition, the expression of the potassium channel *AKT1* gene (PGSC0003DMG400001066) was induced and *KAT3* genes (PGSC0003DMG400009614, PGSC0003DMG400009648) were down-regulated under salt stress, indicating that these proteins have different roles in response to salt in potato (Fig. 10).

### Validation of differential gene expression

**Figure 11:**
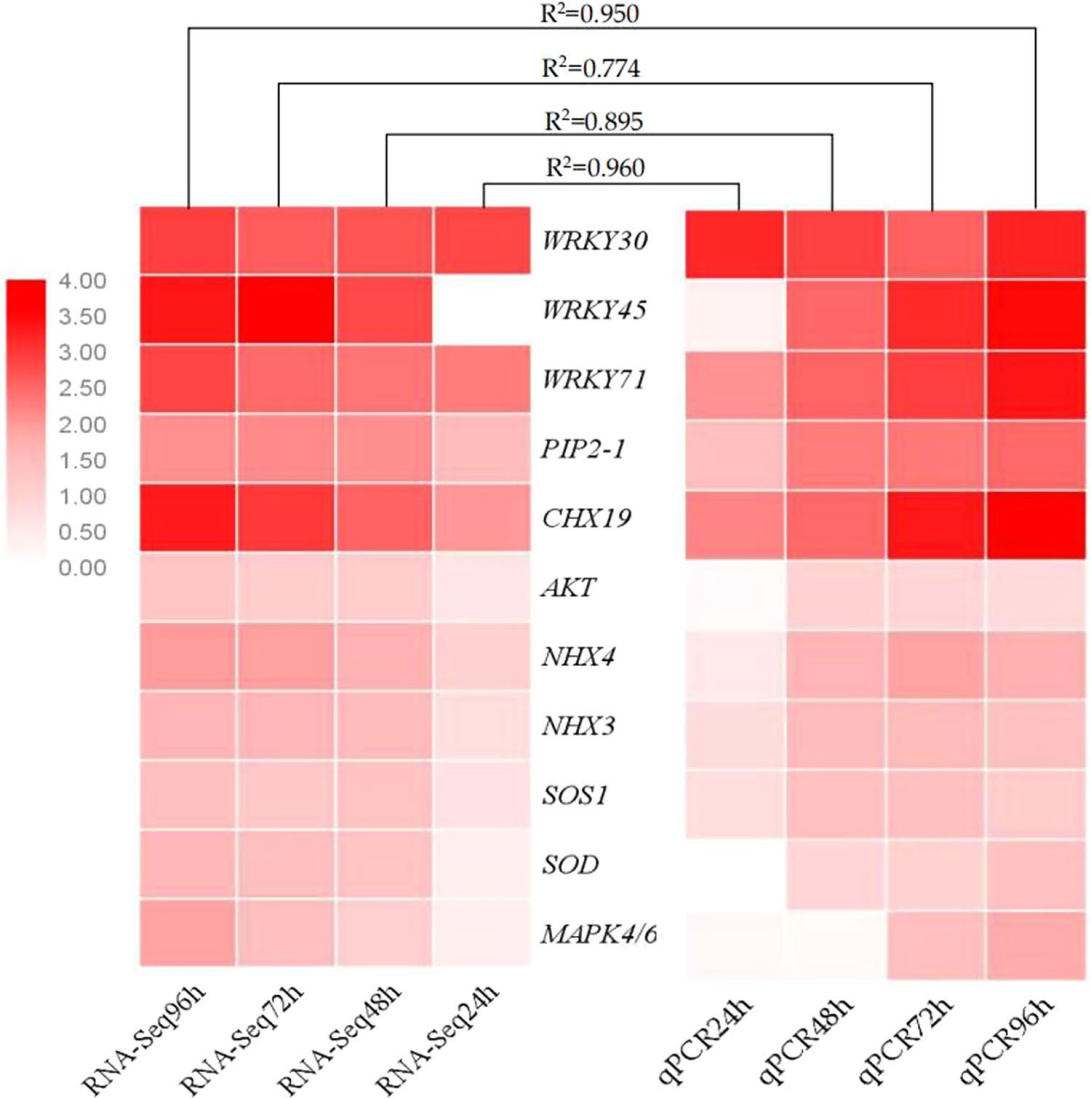
Quantitative real-time PCR analysis of differentially expressed genes. *WRKY*: encodes transcription factor WRKY; *PIP2–1*: encodes aquaporin PIP2–1; *CHX19*: encodes cation/H^+^ antiporter 19; *AKT*: encodes a potassium channel protein; *NHX*: encodes sodium/hydrogen antiporter; *SOS1*: encodes a sodium/hydrogen exchanger; *SOD*: encodes superoxide dismutase; *MAPK4/6*: encodes mitogen-activated protein kinase 4/6.

To validate the differential expression results from transcriptome sequence data analysis, the expression of 11 randomly selected genes differentially expressed under salt stress was evaluated by qRT-PCR. These genes include those encoding transcription factors (WRKY30, WRKY45 and WRKY71), MAPK kinases (MAPK4/6), ion channel proteins (AKT, NHX4, NHX3), CHX19, SOS1, an antioxidant enzyme (SOD) and aquaporin (PIP2-1). The relative expression levels calculated from qRT-PCR correlated with the log2 fold change values determined by RNA-Seq analysis. Thermographic correlation analysis showed that the correlation between relative expression and log2 fold change values at 24 h, 48 h, 72 h and 96 h were very high, with R2 values of 0.96, 0.90, 0.77 and 0.95, respectively (Fig. 11), indicating that the expression levels measured by qRT-PCR were basically consistent with those determined by RNA-seq and that the RNA-seq data were highly reliable.

## Discussion

### The tetraploid potato genotype Longshu No. 5 is an excellent salt-tolerant material

Potato cultivars are highly heterozygous autotetraploids with complicated genetic backgrounds and are moderately sensitive to salt^5^, but there are the evidences that the potatoes have basis for salt tolerance^28,29^. Hence, further research to reveal the salt-tolerance mechanisms in potato is of profound significance for enhancing salt tolerance of potato. The tetraploid potato genotype Longshu No. 5 was obtained from screening an extensive collection of germplasm resources. No significant differences were noted in plant height and fresh weight for the salt-stressed Longshu No.5, indicating that the genotype has strong salt tolerance^23^. In addition, the genetic pedigrees of Longshu No. 5 and Qingshu No. 9 were quite different. It is uncertain if the transcriptome of the two genotypes can be directly compared to obtain the specific salt stress response genes in the salt-tolerant genotype. Therefore, in this study, Longshu No. 5 was subjected to NaCl-induced salt stress, and RNA-seq revealed that a large number of genes encoding TFs, PKs, stress-induced proteins, antioxidation proteins, ion transport proteins and Ca signaling-related proteins were differentially expressed. Meanwhile, many of the identified osmoregulation and antioxidant-related genes (Fig. 7 and Fig. 8) may be contributing to prevent lodging and wilting of potato plants (Fig. 1a). The results demonstrate that these differentially expressed genes have a strong salt-stress response in Longshu No.5.

### The regulatory role of transcription factors in salt tolerance

TFs are important components of plant signal transduction pathways during stress and activate the expression of downstream targets through specific binding to cis-acting elements (CREs)^30^. TFs and their CREs are not only molecular switches for gene expression, but also the termini of signal transduction pathways. WRKY, AP2/ERF, MYB, ZIP, NAC and HSF are well known TF families involved in the response to salt stress^31–38^. For example, a complex containing OsWRKY71, OsMYB1 and OsDOF18 was found to bind and regulate OsRGLp2 (root germinating protein) under low temperature, high temperature, drought and salt stress^39^. In the present study, 274 (5.0%) of the 5508 genes differentially expressed under salt stress encoded TFs. These genes mainly belonged to 13 TF families and zinc finger, AP2/ERF, MYB, bHLH, ZIP, WRKY and NAC included lots of DEGs. Among the TFs identified as being differentially expressed in this study, several have been deemed to play roles in salt stress. For example, the expression levels of *StWRKY7* and *StWRKY14* were previously considered to be significantly up-regulated under salt stress^40^, and OsWRKY45 was reported to enhance tolerance to salinity in plants^41^. In our study, *WRKY45* (PGSC0003DMG400020206) was up-regulated by 11.34-fold at 72 hours. In addition, we found that *WRKY4*, *WRKY29*, *WRKY40*, *WRKY48* and *WRKY61* were differentially regulated, implying that WRKY members might participate in regulating the response to salt stress in potato.

ERF TFs contain a conserved AP2⁄ERF DNA-binding domain^42^ and have been reported to regulate plant development and responses to biotic/abiotic stimuli including drought, salt and cold^26^. DREB1A binds to the C-repeat/DRE element and mediates high salinity-and dehydration-inducible transcription and confers resistance to salt, cold and drought stress in rice^43^. In this study, 23 of 38 ERF TFs were up-regulated under salt stress with DREB 2A-like proteins having expression changes of more than 9-fold, indicating that these proteins positively regulate the response to salt stress in potato.

The MYB domain family is one of the largest TF families, and members play regulatory roles in defense responses in plants^44^. Transgenic potato plants expressing IbMYB1, a sweet potato transcription factor, had enhanced salt stress tolerance and produced high amounts of secondary metabolites^45^. *MYB108*, which was up-regulated with salt stress in the present study, was previously reported being involved in the response to salt stress in *Arabidopsis*^46^. In addition, several MYB TFs, including *LUX*, *AS1* and *DIVARICATA*, were found to be down-regulated under salt stress in our study, demonstrating that MYBs may positively or negatively regulate salt stress response in potato.

Basic leucine zipper (bZIP) TFs play key roles in salt stress signaling^47,48^. *AtbZIP1* is associated with ABA signal transduction and is a positive regulator of plant tolerance to salt^36^. The potato bZIP TF StABF1 is induced in response to salt, drought and ABA stress^48^. The *Agvip1*, a bZIP TF, was up-regulated under salt stress in celery (*Apium graveolens*) cultivars^49^. We found that *VIP*1 (PGSC0003DMG400000799) was up-regulated at salt stress, which indicates that it may play an important role in response to salt stress in potato.

NAC proteins comprise a family of plant-specific TFs that are involved in the regulation of responses to diverse stresses, such as high salt, low temperature and drought^7^. Overexpressing a NAM, ATAF, and CUC (NAC) TF was considered to enhance salt tolerance in rice^50^. Some potato NAC TFs are only induced by salt stress, for example, *StNAC024*, *StNAC067* and *StNAC108*. However, other NAC TFs such as StNAC030, which was up-regulated with salt stress in our study, were previously found to be induced under salt, mannitol and heat treatments^20^. A number of NAC TFs with up-regulated expression under salt stress were identified in this study revealing the potentially crucial role of NAC TFs in potato in salt tolerance.

In summary, zinc finger proteins, ERF, MYB, bHLH, ZIP and WRKY transcription factors responded better to salt stress. Therefore, we should focus on the relationship between these transcription factors and the upstream and downstream genes in response to salt stress in future potato studies.

### Signal transduction-related proteins play a crucial role in the response to salt stress

#### Protein kinases

PKs, which include RLKs, MAPKs, CDPKs and CIPKs, play a key role in the response to salt and other abiotic stresses by sensing external signals and activating signal transduction pathways via phosphorylation of downstream genes^3^. RLKs, which form a large gene family in plants, convey signals to their target proteins in the cytoplasm by phosphorylating them under salt stress^51^. Recent studies have proven that OsRLCK253 (receptor-like cytoplasmic kinase 253)^52^, RPK1 (receptor-like protein kinase 1)^53^, and SRLK (S-receptor-like kinase)^51^ can be induced by salt stress or confer salt tolerance when overexpressed. In addition, RLKs have been shown to activate MAPK cascades and calcium signaling during abiotic stress^54^.

MAPKs play a pivotal role in plant stress resistance signal transduction. There are three kinds of serine/threonine protein kinases involved in MAPK signaling: MAPKKKs, MAPKKs and MAPKs, which transmit and amplify signals through a cascade reaction^55^. It has been shown in many plants that MAPKs can mediate the response to salt stress. In Arabidopsis, overexpression of active MKK9 protein enhances the sensitivity of transgenic seedlings to salt stress, whereas loss of MKK9 activity reduces salt sensitivity^56^, indicating MKK9 negatively regulates the response to salt stress. *MAPK4*, which was up-regulated in this study, was previously found to be highly expressed under salt conditions^57^. In addition, there are reports that *AtMKKK4/6, ZmMKK1, GhMKK3, ZmMPK4, ZmMPK6/7* and *SIMPK3/7* participate in the response to salt stress stimuli^58–62^. In this study, among 49 DEGs involved in the MAPK signaling pathway, 32 genes (including *MAPKK9*, *MAPK4* and *MAPK6*) were up-regulated and 17 (including *NPK1*, *YODA* and *MAPKK6*) were down-regulated. Transcription factor VIP1, which is also involved in the MAPK signaling pathway, can be induced by salt stress^31,49^ and was up-regulated in potato under salt stress. In our study, the differential expression of MAPK-related genes demonstrates that signal cascade is stimulated by salt stress in Longshu No.5.

#### Ca signaling pathway-related proteins

The extracellular stress signal is first perceived by membrane receptors, which then activate complex intracellular signaling cascades that include the generation of Ca^2+^ second messengers. Then Ca^2+^ initiates stress tolerance signaling pathways, and these signals were processed based on the detection and decoding of Ca^2+^ sensors^63^. The Ca^2+^ sensors mainly include CBLs, CMLs, CDPKs, and CIPKs^64^. CIPK serine-threonine protein kinases interact with CBL proteins forming CIPK-CBL complexes, which play an essential role in the response to salt^65,66^. *CBL1* (also known as *SCABP5*) acts as a positive regulator of salt stress responses^67^, and *CBL10* (also known as *SCABP8*) interacts with the *CIPK24* to protect *Arabidopsis* shoots from salt stress^68^. In addition, CIPK21, CIPK25 and CIPK31 regulate plant response to salt stress^69–71^. The CML genes *CML9*, *CML37, CML38* and *CML39* can be induced by salt^72,73^. Plants overexpressing *CPK*, which belongs to a subclass of CDPKs, showed enhanced salt tolerance with an increase in proline and decrease in malonaldehyde^74^. *ZmCPK12* and *OsCPK21* have been reported to confer tolerance to salt stress^75,76^. Twelve of the DEGs in this study encoding Ca^2+^ sensors (*CBL1, CBL10, CML19, CML44, CRCK2, CBP60C, CPK11, CIPK3, CIPK11, CIPK14, CIPK18*, and *CIPK24*) were identified in response to salt stress, demonstrating that Ca signal transduction pathways are activated to mitigate the effects of salt stress in potato.

In the present work, we detected many signal transduction-related genes, especially the differential expression of protein kinase-related genes, MAPK-related genes and Ca signal-related genes. This reveals that various signal transduction pathways are triggered by salt stress in potato.

### Salt stress induces the expression of stress-related genes

Salt stress induces osmotic stress and oxidative stress in plants^77^. In this study, a total of 158 genes encoding osmoregulation, carbohydrate metabolism, and redox regulation related proteins were differentially expressed in response to salt stress. These genes are reported to resist or tolerate the abiotic stress^78–80^. For instance, under salt stress, compatible osmolytes are accumulated, these molecules are important for stabilizing biological structures^81^. In the study, encoding stress-related protein genes were found to be differentially expressed. These DEGs mainly encode PRPs, HSPs, LEA proteins, chaperone proteins, and LTSRs. Proline plays an irreplaceable role in osmotic adjustment. Under stress, proline protects membranes from damage and stabilizes protein structures, delta1-pyrroline-5-carboxylase synthase (P5CS), which is a key enzyme involved in proline synthesis, is induced by salt stress^82^. However, three PRP genes were identified to be down-regulated in our study, showing their distinctive roles in response to salt stress in potato. HSP proteins participate in plant responses to high salt^83,84^, and in the present study, five of six DEGs encoding HSP proteins were up-regulated under salt stress. LEA proteins can improve the resistance of plants to abiotic stress^85^, and *HVA1, OsLEA3* and *LEA14* have been shown to confer tolerance to salt stress^86–88^. A gene (PGSC0003DMG400011437) encoding a LEA protein was found to be up-regulated in this study with a change in expression of 5.59-fold at 24 hours. The differential expression of these stress-related genes in present study show the significance of them in protecting potato from salt-stress damage.

Carbohydrate metabolism plays a major role in salt-induced osmotic regulation^89^. UDP-glycosyltransferases are responsible for transferring sugar moieties onto a variety of small molecules, and contribute to the adaptation of abiotic stress^90^. Overexpression of *UGT79B2/B3* significantly enhanced plant tolerance to low temperatures as well as drought and salt stress^91^ and ectopic expression of a stress-inducible glycosyltransferase (UGT85 family) from saffron enhanced salt and oxidative stress tolerance in *Arabidopsis*^92^. Twelve UDP-glycosyltransferase genes, including *UGT73D1* and *UGT91C1*, were induced by salt stress in this study, indicating that genes involved in carbohydrate metabolism may play crucial roles in salt tolerance in potato.

Salt stress is associated with the rapid production of ROS in plants, and ROS trigger two main antioxidant defense systems (antioxidant enzymes and non-enzyme systems) for protecting cells and scavenging ROS^93^. Transcriptome sequencing of *Pohlia nutans* under salt stress indicated that expression of genes enoding antioxidant enzymes such as CAT, SOD, and POD was enhanced after salt treatment^79^. In this study, genes encoding POD (6 members), SOD (1 member) and CAT (1 member) proteins were up-regulated in response to salt stress. Glutathione S-transferase (GST), an important non-enzyme antioxidant, plays a role in cellular detoxification and stress tolerance. In potato the *StGST* genes are mainly repressed in response to abiotic stresses and induced in response to biotic stress^94^. However, all eight differentially expressed GST genes were up-regulated in the present study. This may be explained by differences in the genetic backgrounds of the salt-tolerant genotype used in this study and the genotype used in the previous study. Furthermore, we found that some genes encoding redox regulation-related proteins in the oxidative phosphorylation pathway, such as cytochrome C oxidases (*COX17, COX6B*) and cytochrome reductases (*ISP, Cyt1, QOR6* and *QCR9*), were up-regulated, indicating that these genes may play positive roles in salt tolerance in potato.

The stress-related genes induced by salt stress may be helpful for maintaining secondary metabolic balance. In particular, the green and spreading leaves of Longshu No.5 exposed to salt stress may be associated with osmotic regulation and oxidation resistance. In addition, we suggest that the oxidative phosphorylation pathway may be involved due to the positive effects of its genes in response to salt stress in potato.

### The potential molecular mechanisms underlying the response to salt stress in potato

High concentrations of salt (mainly Na^+^) in plant cells can induce ionic stress. Therefore, initiating the re-establishment of cellular ionic homeostasis under stress conditions is crucial^12^. SOS is a Ca^2+^ signal-dependent plant salt stress response pathway^95^, SOS2 is activated under salt stress, and phosphorylates and activates SOS1, which is a key element involved in Na^+^ transport from the cytoplasm to the apoplast^96^, and NHX, a vacuolar Na^*+*^/H^*+*^ exchanger^97^ for the purpose of ionic equilibrium. A gene (PGSC0003DMG400006384) encoding SOS2 was up-regulated in this study, indicating that this gene plays a positive regulatory role in the response to salt stress.

In our study, many genes related to ANN–14-3-3–PKS5/24/J3–SCaBP5/8–SOS2–SOS1 and ANN–14-3-3–PKS5/24/J3–SCaBP5/8–SOS2–NHXs signaling pathway were found to be differentially expressed. The genes encoding 14-3-3 proteins (PGSC0003DMG400017753, PGSC0003DMG400023590, PGSC0003DMG400012899), CBL10/SCaBP8 (PGSC0003DMG400029942), J3 (PGSC0003DMG400024784), CBL1/SCaBP5 (PGSC0003DMG400020493), CIPK11/PKS5 (PGSC0003DMG400020564), and CIPK14/PKS24 (PGSC0003DMG400022019, PGSC0003DMG400011106) were up-regulated in response to salt stress and ANNs (PGSC0003DMG402019427, PGSC0003DMG400019446, PGSC0003DMG400001879) were down-regulated. ANNs mediates early transient Ca^2+^ signal production, so the down-regulation of ANN may be beneficial for maintaining the intracellular Ca^2+^ concentration^14^. In addition, we found that PGSC0003DMG400022490, PGSC0003DMG400010663, PGSC0003DMG401021988 and PGSC0003DMG402021988, which encode NHX proteins; PGSC0003DMG400010630, which encodes SOS1; 13 V-ATPase genes and 3 PM-ATPase genes were up-regulated under NaCl stress, indicating that they may play a crucial role in maintaining the level of Na^+^ in the cytoplasm under salt stress in potato.

To date, the complete SOS pathway has yet to be established for salt-stress response in potato, and only a few genes of this pathway have been reported. Many SOS-related genes have been identified in our study, providing strong evidence that an SOS pathway mediates tolerance to salt stress in potato.

## Materials and Methods

### Plant materials

In a previous study, we screened 52 potato varieties for salt tolerance and found that Longshu No. 5 exhibited excellent growth and strong salt tolerance^23^. Specifically, the 25-day seedlings were cut into single stem segments, and the small segments were inoculated into Murashige and Skoog (MS) medium with the presence and absence of NaCl, with three replicates of 5 plants per replicate. After 4 weeks, the plant height, total biomass and fresh weight were measured. For Longshu No. 5, there was no significant difference in plant height between salt-stress and control conditions. Compared with the controls, there was a significant increase in fresh weight under salt stress. However, the height and fresh weight of Qingshu No. 9 samples were significantly decreased under salt stress compared with controls. Due to the fact that cultivated potato is an autotetraploid with a complex genetic background, the genetic pedigrees of Longshu No. 5 and Qingshu No. 9 were quite different. Meanwhile, considering the high cost of sequencing, we focused on the salt-stress response genes in salt-tolerant materials. Therefore, in this study, transcriptome analysis was only performed for the salt-tolerant genotype Longshu No. 5 under salt stress.

### NaCl-induced salt stress

Seedlings of Longshu No. 5 were grown on normal MS medium for 4 weeks, then seedlings with the same shape, were selected. The stem segments with 7 ± 1 stem nodes were cut and inserted into MS medium containing 0 mmol/L or 500 mmol/L NaCl; 5 stem segments were inserted in each replicate with three replicates per treatment. Next the seedlings were cultured at (20 ± 2) °C with 16 hours illumination each day. At 0 (samples without treatment), 24, 48, 72 and 96 hours, plant samples were harvested from three replicates per treatment, immediately frozen in liquid nitrogen, and stored at −80 °C.

### RNA sequencing

Total RNA was extracted from frozen samples using Trizol reagent (Invitrogen, Carlsbad, CA, USA), and then purified using the RNeasy Plant Mini Kit (Qiagen, Valencia, CA, USA) according to the manufacturer’s protocol. RNA integrity was determined using a 2100 Bioanalyzer (Agilent) and quantified using a NanoDrop (Thermo Scientific). RNA samples (OD260/280 = 1.8 to 2.2, OD260/230 ≥ 2.0, RIN ≥ 8, >500 ng) were used for constructing sequencing libraries. The libraries were prepared using the NEBNext UltraTM RNA Library Prep Kit (E7530L) for Illumina (NEB, USA). Next, Qubit 2.0 fluorometer dsDNA HS Assay (Thermo Fisher Scientific) was used to measure concentration of the resulting sequencing libraries, while the size distribution was analysed using an Agilent BioAnalyzer 2100. Finally, the libraries were sequenced on the Illumina HiSeq X Ten platform for generating 150-bp paired-end reads.

### Data quality control and mapping clean reads to the genome

After sequencing, raw data were saved in FASTQ format. Raw reads were subjected to quality filtering using the NGS QC Toolkit v2.3.3^98^. To obtain clean reads, the reads containing adapter or poly-N, and other low-quality reads were removed. The high-quality clean data were used for downstream data calculation and analyses, such as Q20, Q30 and GC-content. The clean reads were mapped back onto the reference genome sequence (PGSC_DM_v4.03) from the genotype of *S. tuberosum* group Phureja DM1-3 516 R44 (here referred to as DM), and were aligned using Tophat2 (v2.0.13)^99^. Meanwhile, we downloaded annotation files from the ENSEMBL plants database (ftp://ftp.ensemblgenomes.org/pub/plants/release-34/fasta/solanum_tuberosum/)^100^. Base on the reference genome and perfect-match or one-mismatch reads, we performed further annotation and analyses.

### Differential expression analysis and enrichment analysis of DEGs

The expression values from the Illumina reads for each sample were determined with RSEM using default parameters^101^, Reads per kilobase of exon model per million mapped reads (FPKM) was calculated to estimate gene expression levels using HTseq-count^102^. The FPKM threshold value was set at 0.1. Differential expression analysis for each comparison was performed using DESeq. 2 from three biological replicates^103,104^. The adjusted P-values were used to control the false discovery rate (FDR). Genes with a corrected P-value < 0.05, fold-change ≥ 2 and FDR ≤ 0.01 were considered as DEGs. Pairwise comparisons were performed between each HS group (24 h, 48 h, 72 h and 96 h) and the respective CK group to obtain a salt-stress group (HS) DEG library, and the CK groups (0 h, 24 h, 48 h, 72 h and 96 h) were compared with each other to obtain a control group (CK) DEG library. In total, 4297 and 5558 DEGs were found from the CK groups and HS groups, respectively. The total salt-stress response DEGs were obtained by removing genes in the CK-DEG library from the HS-DEGs library.

To determine which category DEGs belonged to, we performed GO enrichment analysis using the GOseq R package. The technique applies the non-central hyper-geometric test to correct gene length bias^105^. KEGG enrichment was performed using KOBAS 2.0 to reveal significantly enriched signal pathways in DEGs^106^. GO terms and KEGG pathways with an adjusted P-value < 0.05 were considered to be significantly enriched in DEGs.

### Quantitative real-time PCR validation

To verify RNA-seq results, qRT-PCR was performed for 11 randomly selected DEGs (Supplementary Table S8). The samples used for RNA-seq and qRT-PCR analysis were the same ones. The qRT-PCR protocol used here is described in our previous study^26^. The experiment was performed using a Roche LC480 II System (Roche Diagnostics Nederland BV, Almere, the Netherlands) with three technical and three biological replicates. The 2^**−∆∆Ct**^ method^107^ was used to calculate the relative expression level of each gene using the internal reference gene *actin*. All primers used in this study (Supplementary Table S8) were designed using Primer 5.0.

Data availability The datasets generated and/or analysed during the current study are available from the corresponding author on reasonable request. Raw RNA-seq data from the study have been deposited in the NCBI SRA (sequence read archive) database under accession PRJNA577000.

## Supplementary information

Supplementary Information.
