# Supplementary material for: Transcriptome analysis uncovers the gene expression profile of salt-stressed potato (Solanum tuberosum L.)
Source: Sci Rep. 2020 Mar 25;10:5411. doi: 10.1038/s41598-020-62057-0 (PMC7096413; doi:10.1038/s41598-020-62057-0)
Supplement: Supplementary file 1 — Supplementary Information. [file 41598_2020_62057_MOESM1_ESM.pdf]

Supplementary Information

Manuscript title：Transcriptome analysis uncovers the gene expression profile of salt-stressed potato (*Solanum tuberosum* L.)

Author list：Qing Li[1,2], Yuzhi Qin[2], Xinxi Hu[2], Guangcun Li[1], Hongying Ding[2], Xingyao Xiong[1,2,*] & Wanxing Wang[1,*]

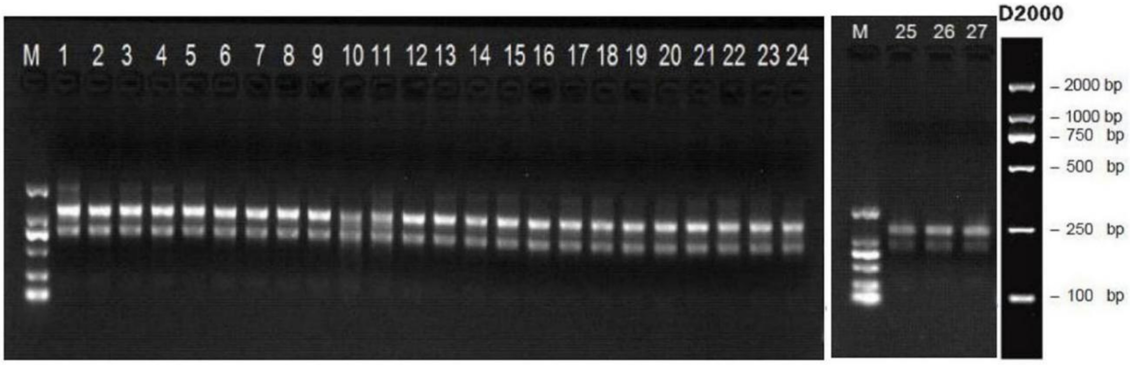

Figure S1. Gels electrophoretogram of RNA samples used for RNA-seq. Lane M: D2000 DNA marker. Total 27 samples and two gels. Lanes 1-24: CK_24h_rep1; CK_24h_rep2; CK_24h_rep3; CK_48h_rep1; CK_48h_rep2; CK_48h_rep3; K_72h_rep1; CK_72h_rep2; CK_72h_rep3; CK_96h_rep1; CK_96h_rep2; CK_96h_rep3; HS_24h_rep1; HS_24h_rep2; HS_24h_rep3; HS_48h_rep1; HS_48h_rep2; HS_48h_rep3; HS_72h_rep1; HS_72h_rep2; HS_72h_rep3; HS_96h_rep1; HS_96h_rep2; HS_96h_rep3. Lanes 25-27: CK_0h_rep1; CK_0h_rep2; CK_0h_rep3.

Table S1. Quality of the RNA samples used for RNA-seq.

Sample name	Volume (µl)	Nanodrop check			Qubit c (ng/µl)	RIN	28s/18s
		C (ng/µl)	260/280	260/230			
CK_0h_rep1	32	1164.5	2.15	1.64	1076.4	8.3	1.9
CK_0h_rep2	32	1258.1	2.16	1.83	1209.0	8.3	1.8
CK_0h_rep3	33	1189.3	2.15	1.65	1030.4	8.6	1.9
CK_24h_rep1	34	846.7	2.14	1.66	710	8.5	1.9
CK_24h_rep2	34	1280.4	2.16	1.49	1267.2	8.6	2
CK_24h_rep3	34	1935.8	2.16	2.31	1728	8.3	2
CK_48h_rep1	35	1043.7	2.15	1.4	1044.4	8.5	1.9
CK_48h_rep2	35	1031.8	2.17	0.81	1041.6	8.5	2
CK_48h_rep3	34	1976.5	2.17	2.19	1540.8	8.4	2
CK_72h_rep1	34	1367.9	2.16	2.08	1210.4	8.3	2
CK_72h_rep2	34	1599.2	2.17	1.82	1356.6	8.2	1.9
CK_72h_rep3	40	1141.4	2.15	1.68	1008	8.2	1.8
CK_96h_rep1	33	1217.9	2.16	1.62	1107	8.3	2
CK_96h_rep2	35	1419.7	2.17	2.1	1179.8	8.3	2
CK_96h_rep3	35	1152.3	2.17	2.12	800.8	8	1.8
HS_24h_rep1	36	1000.2	2.17	1.82	832.8	8.6	2.3
HS_24h_rep2	34	1565.1	2.15	2.25	1352.8	8.4	1.9
HS_24h_rep3	34	1433.4	2.16	2.43	1166.2	8.5	2
HS_48h_rep1	34	2012.3	2.17	1.67	1835	8.7	2.2
HS_48h_rep2	34	1247.9	2.16	1.97	1086	8.1	1.8
HS_48h_rep3	34	954.8	2.16	1.54	796.4	8.5	1.8
HS_72h_rep1	34	1045.1	2.16	2.27	930.8	8.3	1.9
HS_72h_rep2	32	1173.9	2.15	2.1	971.6	8.5	2
HS_72h_rep3	32	1028.7	2.16	2.32	873.6	8.7	2.2
HS_96h_rep1	34	1561.6	2.16	1.81	1386	8.6	1.8
HS_96h_rep2	34	1235.3	2.15	2.46	1213.8	8.4	1.9
HS_96h_rep3	34	1054	2.16	1.74	999	8.6	2

Table S2. Basic summary of RNA-sequencing results.

Sample	Clean reads	Clean Bases (G)	Q30 Rate	Mapped Reads	Mapping Rate
CK_0h_rep1	68,786,334	10.30	96 %	59,059,337	85.9%
CK_0h_rep2	74,622,816	11.17	96%	63,726,752	85.4%
CK_0h_rep3	76,832,112	11.50	96%	65,651,239	85.5%
CK_24h_rep1	91,564,176	13.71	96%	78,175,813	85.4%
CK_24h_rep2	63,474,958	9.50	96%	54,054,249	85.2%
CK_24h_rep3	70,728,492	10.59	96%	60,023,862	84.9%
CK_48h_rep1	80,061,110	11.98	96%	67,724,529	84.6%
CK_48h_rep2	80,570,890	12.06	96%	67,966,958	84.4%
CK_48h_rep3	91,018,958	13.63	96%	77,060,433	84.7%
CK_72h_rep1	71,213,164	10.66	96%	60,759,056	85.3%
CK_72h_rep2	91,224,096	13.64	96%	77,963,250	85.5%
CK_72h_rep3	76,989,946	11.52	96%	65,848,603	85.5%
CK_96h_rep1	77,672,948	11.63	96%	66,125,277	85.1%
CK_96h_rep2	55,819,120	8.36	95%	47,298,962	84.7%
CK_96h_rep3	72,221,390	10.81	95%	60,972,079	84.4%
HS_24h_rep1	77,033,804	11.53	96%	65,659,336	85.2%
HS_24h_rep2	82,306,006	12.32	96%	70,382,837	85.5%
HS_24h_rep3	76,596,384	11.47	96%	65,331,065	85.3%
HS_48h_rep1	80,502,446	12.05	96%	69,328,019	86.1%
HS_48h_rep2	68,241,212	10.22	96%	58,389,090	85.6%
HS_48h_rep3	83,523,264	12.50	96%	71,911,611	86.1%
HS_72h_rep1	85,161,088	12.75	96%	73,397,071	86.2%
HS_72h_rep2	98,028,906	14.66	97%	84,060,341	85.8%
HS_72h_rep3	81,785,110	12.24	96%	69,845,492	85.4%
HS_96h_rep1	67,237,994	10.07	95%	57,645,822	85.7%
HS_96h_rep2	80,113,982	11.98	96%	68,498,847	85.5%
HS_96h_rep3	79,803,548	11.94	96%	68,275,727	85.6%

Table S3. Main Kyoto Encyclopedia of Genes and Genomes (KEGG) pathways enriched in differentially expressed genes in Longshu No .5.

Pathway Terms	Map ID	Number of DEGs	Q-value
Photosynthesis	map00195	35	8.71E-13
Carbon fixation in photosynthetic organisms	map00710	40	3.89E-10
Citrate cycle (TCA cycle)	map00020	31	9.91E-10
Glyoxylate and dicarboxylate metabolism	map00630	40	7.07E-09
Spliceosome	map03040	10	1.33E-05
Ribosome	map03010	37	0.000659363
Phenylalanine, tyrosine and tryptophan biosynthesis	map00400	22	0.001669819
Valine, leucine and isoleucine degradation	map00280	27	0.001914245
Pentose phosphate pathway	map00030	21	0.003589959
Oxidative phosphorylation	map00190	44	0.006039445
Propanoate metabolism	map00640	20	0.007766872
Ribosome biogenesis in eukaryotes	map03008	5	0.011337509
Pentose and glucuronate interconversions	map00040	11	0.016441266
RNA transport	map03013	15	0.018742182
Pyruvate metabolism	map00620	31	0.018742182
Glycine, serine and threonine metabolism	map00260	25	0.025919038
Phagosome	map04145	26	0.045743129
MAPK signaling pathway - plant	map04016	49	0.476642346

Table S4. Transcription factors differentially expressed under salt treatment.

Transcription factor families	Number	Known genes*	
		up	down
Zinc finger	60	*SAP13, SAP12, ZFWD1, ZAT2, ZNF511*	*ZAT5, DOF3.4, DOF3.6,GATA4, GATA19*
AP2/ERF	38	*AIL1, ERF114, ERF1, ERF020, SHINE3*	*ERF12, ERF023, RAP2-7, TINY, AIL5, AIL7, ANT, RAP2-13, WIN1, SlAP2e*
MYB	37	*YOR301W, AIM1, RAX2, MYB108, MYB36, MYB122*	*AS1, LUX, DIVARICATA*
bHLH	33	*BHLH41, BHLH47, BHLH66, BHLH69, BHL93, BHLH140, BHLH157, SPCH, DYT1, SPT*	*BHLH35, FAMA*
ZIP	25	*bZIP43, bZIP53, TGA-2.1, HAT5, VIP1*	*ATHB-6, bZIP27, HAT4, HAT5, GLABRA 2*
WRKY	23	*WRKY4, WRKY6, WRKY7, WRKY14, WRKY29, WRKY40, WRKY45, WRKY48, WRKY61,WRKY71*	*WRKY2, WRKY6, WRKY21, WRKY30, WRKY44, WRKY51, WRKY70*
NAC	11	*NAC030, NAC083*	*NAC031*
TCP	11	*TCP17*	*TCP12, TCP14, TCP19*
Others	11	--	--
HSF	7	*HSF8, HSFB4*	*HSFC1, HSB3*
Homeobox TFs	5	*AP2, BLH1, LET12*	*ATH1*
NFY	5	--	--
ARF	4		*ARF4, ARF5, ARF9*
MADS	4	*AGL15*	

--: no known function in other plants. *: only genes with known functions in other plants are listed here. HAT5 and WRKY6 each had two genes with opposite expression that were induced by salt stress in potato.

Table S5. Protein kinase genes differentially expressed under salt stress.

Protein kinases	Number	Known genes*
LRR receptor-like kinases, LRR-RLKs	46	*ERECTA, ERL1, FEI2, GSO1, IRK, RKF3, SOBIR, TDR, BAM1, BAM3, IMK2*
Receptor-like protein kinases, RPKs	26	*HAIKU2, HERK1, HSL1, TMK3, TMK4*
Receptor-like serine/threonine-protein kinases, STKs	17	*CES101, RLK1, SD2-5, SD1-7, SD1-8*
Calmodulin-binding receptor-like cytoplasmic kinases, CRCKs	4	*CRCK2*
Inactive receptor kinases, IRKs	4	--
LysM domain receptor-like kinases, LYKs	4	*LYK3, LKY4*
Wall-associated receptor kinases-like, WAKLs	4	*WAKL2, WAKL14, WAKL16, WAKL20*
Chitin elicitor receptor kinases, CERKs	3	--
Receptor-like cytosolic protein kinases, RLCKs	3	*RBK1, RBK2*
Cysteine-rich receptor-like protein kinases, CRKs	2	*CRK2, CRK3*
L-type lectin-domain containing receptor kinases, LRKs	2	*LECRKS1, LECRK71*
Proline-rich receptor-like protein kinases, PERKs	2	*PERK9, PERK13*
BRASSINOSTEROID INSENSITIVE 1-associated receptor kinases, BAKs	6	*BAK1*
Somatic embryogenesis receptor kinases, SERKs	1	*SERK1*
Serine/threonine protein kinases, STPKs	47	*BLUS1, CDL1, KIPK, NAK, STN8, WNK10, WNK11, WNK4, WNK5, ACR4, CCR3, STPK1*
CBL-interacting protein kinases, CIPKs	7	*CIPK2, CIPK3, CIPK11, CIPK14, CIPK18*
Mitogen-activated protein kinase kinase kinases, MAPKKKs	5	*NPK1, YODA*
Adenylate kinases, AKs	3	*AK4*
Inactive protein kinases, IPKs	3	--
Inositol-tetrakisphosphate kinases, ITPKs	3	--
Pyruvate kinases, PYKs	4	*PKP2*
Shaggy-related protein kinases, KSGs	3	*ASK5, ASK7, ASK10*
ATP-dependent 6-phosphofructokinases 5, PFKAs	2	--
Xylulose kinases, XKs	2	--
Phosphoenolpyruvate carboxylase kinases, PPCKs	2	--
Calcium-dependent protein kinases, CDPKs	2	*CDPK1*
Shikimate kinases like, SKLs	2	*aroL*
Chloroplast sensor kinases, CSKs	1	--
Cyclin-dependent kinases, CDKs	1	*CDK7*
D-glycerate 3-kinases, GLYKs	1	--
Fructokinases, FRKs	1	--
Glycerol kinases, GKs	1	*glpK*
Casein kinases, CKs	1	--

Inositol 3-kinases	1	--
Inositol hexakisphosphate and Diphosphoinositol-pentakisphosphate kinases, PPIP5Ks	1	*VIP*
Inositol polyphosphate multikinases, IPKs	1	*IPK2*
Non-functional pseudokinases, NFPKs	1	*ZED*
Pantothenate kinases, PANKs	1	*PANK*
Adenosine kinases,ADKs	1	*ADK2*
Phosphoenolpyruvate carboxykinases, PEPCKs	1	*pckA*
Phosphoglycerate kinases, PGKs	1	*pgk*
phosphoribulokinases, PRKs	1	*prkB*
Protein NSP-INTERACTING kinases, NIKs	1	-
Protein-ribulosamine 3-kinases, FN3KRPs	1	-
PTI1-like tyrosine-protein kinases, PTIs	1	*PTI1*
Pyruvate dehydrogenase (acetyl-transferring) kinases, PDKs	1	-

--: no known function in other plants. *: only genes with known functions in other plants are listed here.

Table S6. MAPK signal pathway genes differentially expressed in response to salt stress in potato.

Gene ID	log2 Fold Change				Description
	24 h	48 h	72 h	96 h	
PGSC0003DMG400017864	-3.37	-6.08	-6.88	-8.51	receptor-like protein kinase
PGSC0003DMG400000193	-4.57	-6.35	-8.35	-8.28	1-aminocyclopropane-1-carboxylate synthase 2
PGSC0003DMG400033565	-0.90	-3.49	-5.28	-7.63	calmodulin-5/6/7/8-like
PGSC0003DMG400014594	-2.79	-4.95	-5.81	-5.51	ethylene-responsive transcription factor 1B-like
PGSC0003DMG400017186	-0.35	-1.48	-3.60	-4.08	ethylene receptor 2-like
PGSC0003DMG400001529	-0.81	-2.09	-3.05	-3.74	acidic 27 kDa endochitinase
PGSC0003DMG400009906	-0.54	-1.57	-2.65	-3.37	catalase isozyme 3
PGSC0003DMG400006639	-2.61	-2.58	-2.78	-3.37	serine/threonine-protein kinase OXI1-like
PGSC0003DMG400015021	-4.02	-2.92	-4.53	-3.09	mitogen-activated protein kinase kinase kinase A-like
PGSC0003DMG400013402	-	-	-0.61	-3.09	ethylene-responsive transcription factor 1B-like
PGSC0003DMG400000771	-0.36	-1.47	-2.00	-2.96	protein MKS1-like
PGSC0003DMG400015020	-1.54	-	-1.74	-2.89	mitogen-activated protein kinase kinase kinase A-like
PGSC0003DMG400025366	-0.36	-1.14	-1.77	-2.78	MAPK4/6
PGSC0003DMG400022210	-0.75	-0.67	-0.80	-1.72	mitogen-activated protein kinase kinase kinase 2-like
PGSC0003DMG402000057	-0.03	-0.56	-1.28	-1.52	MAPK4_2 protein
PGSC0003DMG400005825	-1.76	-2.81	-1.37	-1.52	1-aminocyclopropane-1-carboxylate synthase
PGSC0003DMG400026748	-0.60	-1.53	-1.48	-1.51	LRR receptor-like serine/threonine-protein kinase
PGSC0003DMG400012276	-0.05	-0.82	-1.26	-1.42	nucleoside-diphosphate kinase
PGSC0003DMG400012594	-0.36	-0.94	-1.29	-1.30	BRASSINOSTEROID INSENSITIVE 1-associated receptor kinase 1-like
PGSC0003DMG400009101	-0.28	-1.97	-0.58	-1.29	transcription factor SPEECHLESS
PGSC0003DMG400008712	-0.44	-0.88	-1.35	-1.27	protein ETHYLENE INSENSITIVE 3-like
PGSC0003DMG400017965	-0.24	-0.63	-1.05	-1.23	calmodulin-like protein
PGSC0003DMG400007843	-0.17	-0.50	-0.86	-1.22	ethylene receptor 1 isoform X1
PGSC0003DMG400003528	-0.24	-0.50	-0.84	-1.19	mitogen-activated protein kinase 7
PGSC0003DMG400015515	-0.21	-0.78	-1.19	-1.13	protein RTE1-HOMOLOG
PGSC0003DMG400000799	-0.16	-0.51	-0.84	-1.09	transcription factor VIP1
PGSC0003DMG400005540	-0.21	-0.60	-1.16	-1.07	calmodulin-2/4-like
PGSC0003DMG400007205	-0.27	-0.71	-1.17	-1.07	calmodulin-like
PGSC0003DMG400030830	-0.38	-0.94	-1.07	-1.05	serine/threonine-protein kinase SAPK2-like
PGSC0003DMG400029941	-0.25	-0.35	-1.04	-0.74	green ripe-like 1
PGSC0003DMG400012316	-0.53	-0.73	-1.52	-0.71	respiratory burst oxidase homolog protein A
PGSC0003DMG400026749	-0.60	-0.89	-1.33	-0.70	LRR receptor-like serine/threonine-protein kinase
PGSC0003DMG402026149	1.34	0.39	3.82	-0.14	mitogen-activated protein kinase kinase kinase 1-like
PGSC0003DMG400033696	0.40	0.53	1.31	0.21	mitogen-activated protein kinase kinase 9
PGSC0003DMG400031028	0.27	0.48	1.22	0.64	LRR receptor-like serine/threonine-protein kinase ERL1

PGSC0003DMG400023441	-0.23	1.46	1.03	0.67	serine/threonine-protein kinase SAPK7-like
PGSC0003DMG400029408	1.12	1.58	1.30	0.81	catalase isozyme 1-like protein
PGSC0003DMG403005720	0.45	1.47	1.13	0.98	mitogen-activated protein kinase kinase 6
PGSC0003DMG400028666	0.22	0.96	0.13	1.18	mitogen-activated protein kinase kinase kinase YODA
PGSC0003DMG400017514	0.39	0.77	1.23	1.19	abscisic acid 8'-hydroxylase 3-like
PGSC0003DMG400018992	0.85	1.70	1.25	1.42	mitogen-activated protein kinase kinase kinase NPK1-like
PGSC0003DMG400018811	0.34	1.10	1.88	1.52	LRR receptor-like serine/threonine-protein kinase
PGSC0003DMG400016156	0.65	1.13	0.58	1.54	serine/threonine-protein kinase SAPK1-like
PGSC0003DMG400012203	-0.10	0.59	0.72	1.79	mitogen-activated protein kinase kinase kinase YODA
PGSC0003DMG400012514	0.73	0.01	0.83	1.95	LRR receptor-like serine/threonine-protein kinase FLS2
PGSC0003DMG400008997	0.79	1.69	2.00	2.13	mitogen-activated protein kinase kinase kinase NPK1
PGSC0003DMG400002028	0.91	0.34	1.64	3.08	pathogenesis-related protein 1b precursor
PGSC0003DMG400002029	1.05	0.30	1.55	3.13	pathogenesis-related protein 1b precursor
PGSC0003DMG400002027	1.09	0.28	1.50	3.25	pathogenesis-related protein 1b precursor

Table S7. Carbohydrate metabolism-related genes differentially expressed in response to salt stress.

Gene ID	log2 Fold Change				Description
	24 h	48 h	72 h	96 h	
PGSC0003DMG400003901	-0.44	-0.85	-0.51	-2.10	UDP-glycosyltransferase 73C1-like
PGSC0003DMG400004659	0.30	2.68	1.80	2.79	UDP-glucuronate 4-epimerase 6-like
PGSC0003DMG400006908	-0.29	-1.67	-1.48	-2.38	UDP-glycosyltransferase 90A1-like
PGSC0003DMG400008600	-	-	-3.96	-6.38	UDP-glycosyltransferase 73D1
PGSC0003DMG400009525	-2.91	-4.09	-3.71	-4.21	UDP-glycosyltransferase 74G1-like
PGSC0003DMG400009527	-2.81	-4.32	-4.13	-4.66	UDP-glycosyltransferase 74G1-like
PGSC0003DMG400009528	-0.88	-2.82	-2.31	-2.66	UDP-glycosyltransferase 74G1-like
PGSC0003DMG400009529	-3.18	-4.01	-3.75	-4.60	UDP-glycosyltransferase 74G1-like
PGSC0003DMG400010168	0.41	2.09	1.13	1.55	UDP-N-acetylmuramate--L-alanine ligase-like
PGSC0003DMG400012012	0.57	1.12	2.33	2.65	fructose-bisphosphate aldolase 1
PGSC0003DMG400013275	-0.19	-0.99	-1.86	-2.01	mannan endo-1,4-beta-mannosidase 6-like
PGSC0003DMG400015437	0.40	1.59	2.10	2.44	UDP-glucose flavonoid 3-O-glucosyltransferase 7
PGSC0003DMG400015579	-3.69	-3.42	-4.27	-5.20	UDP-glycosyltransferase 74B1-like
PGSC0003DMG400020363	0.63	1.14	2.16	2.49	fructose-1,6-bisphosphatase precursor
PGSC0003DMG400021116	-0.88	-2.24	-2.38	-3.13	UDP-glycosyltransferase 91C1
PGSC0003DMG400021694	0.58	1.45	-	2.69	UDP-glycosyltransferase 76C4-like
PGSC0003DMG400024109	1.20	3.20	3.41	4.27	hypothetical protein ZEAMMB73_780929
PGSC0003DMG400024246	-0.92	-1.62	-2.04	-2.28	probable fructokinase-7
PGSC0003DMG400026930	-1.19	-1.93	-1.61	-2.60	UDP-glycosyltransferase 73C3-like
PGSC0003DMG400028361	-0.48	-1.34	-1.52	-2.21	alpha-1,4-glucan-protein synthase [UDP-forming]1
PGSC0003DMG400030454	-1.17	-2.79	-3.43	-3.20	UDP-glycosyltransferase 87A1-like
PGSC0003DMG400030565	-0.17	-1.25	-2.25	-2.58	probable fructose-bisphosphate aldolase 3
PGSC0003DMG401011335	-0.85	-	-1.53	-2.55	UDP-glucuronic acid decarboxylase 4-like

Table S8. Primers used in qRT-PCR.

Genes	Gene-id	Reverse primer	Forward primer
AKT	PGSC0003DMG400001066	ATAGAGCCACTTATCTACTTGT	GAAGCATATTGAATAATCCATACTG
NHX4	PGSC0003DMG400022490	ACAATGTGACCGAGAGTT	ATGCCGACATAGAGGAAG
NHX3	PGSC0003DMG400010663	ATTGGAGATTACCTTGCTATTG	TTGCTTGTGTTGATATGAGATAA
MAPK4/6	PGSC0003DMG400025366	GACGAAGGAGGAGATAGC	CTGTCTGGTGGTCTTACTAT
WRKY71	PGSC0003DMG400009703	CACACTCATCCTATTGACAAG	TGAAGCAACAGAGCAGTA
WRKY30	PGSC0003DMG400009103	CCAACAACTCCTATTATACTCTG	GCTGCGTAATTGTCCATT
WRKY45	PGSC0003DMG400020206	CACTCATCCTATTGACAAGC	TGAAGCAACAGAGCAGTA
CHX19	PGSC0003DMG400012168	GATTGTTGGCACGATTGT	GTGATGAATGTTGTGAAGAGA
SOD	PGSC0003DMG400010660	TCAACAGGACCACATTACA	GTGAACCAGTGAGAGGAA
SOS1	PGSC0003DMG400010630	TGGTATGGATGCTCTATCAG	TTATTGCTTCCACTACAGATTC
PIP2-1	PGSC0003DMG400024197	CCAGCACCATTAGTAGACT	CAAGCAATACCAAGAATACCA
actin	——	CCGATCTCCTCTCAGTTC	CCATTCCAGTTCCATTGTC
